# The First Mitochondrial Genome of *Ciborinia camelliae* and Its Position in the Sclerotiniaceae Family

**DOI:** 10.3389/ffunb.2021.802511

**Published:** 2022-02-09

**Authors:** Irene Valenti, Luca Degradi, Andrea Kunova, Paolo Cortesi, Matias Pasquali, Marco Saracchi

**Affiliations:** Department of Food, Environmental and Nutritional Sciences, University of Milan, Milan, Italy

**Keywords:** fungal diversity, pathogen of *Camellia*, mitochondrial assembly and annotation, short and long read sequencing, mitochondrial diversity

## Abstract

*Ciborinia camelliae* is the causal agent of camellia flower blight (CFB). It is a hemibiotrophic pathogen, inoperculate Discomycete of the family Sclerotiniaceae. It shows host and organ specificity infecting only flowers of species belonging to the genus *Camellia*, causing serious damage to the ornamental component of the plant. In this work, the first mitochondrial genome of *Ciborinia camellia* is reported. The mitogenome was obtained by combining Illumina short read and Nanopore long read technology. To resolve repetitive elements, specific primers were designed and used for Sanger sequencing. The manually curated mitochondrial DNA (mtDNA) of the Italian strain DSM 112729 is a circular sequence of 114,660 bp, with 29.6% of GC content. It contains two ribosomal RNA genes, 33 transfer RNAs, one RNase P gene, and 62 protein-coding genes. The latter include one gene coding for a ribosomal protein (*rps3*) and the 14 typical proteins involved in the oxidative metabolism. Moreover, a partial mtDNA assembled from a contig list was obtained from the deposited genome assembly of a New Zealand strain of *C. camelliae*. The present study contributes to understanding the mitogenome arrangement and the evolution of this phytopathogenic fungus in comparison to other Sclerotiniaceae species and confirms the usefulness of mitochondrial analysis to define phylogenetic positioning of this newly sequenced species.

## Introduction

The genus *Ciborinia* involves 23 different species of host-specific pathogens (https://eol.org/). *Ciborinia* spp. are members of the Sclerotiniaceae family (order Helotiales; phylum Ascomycota), which includes 14 genera of phytopathogenic fungi with a wide host range. The Sclerotiniaceae's ability to infect different hosts and adapt to various environments results in serious economic damage (Bolton et al., 2006). Unlike the other Sclerotiniaceae, *Ciborinia camelliae* Kohn infects only plants of the genus *Camellia* causing the camellia flower blight (CFB) (Taylor and Long, 2000; Saracchi et al., 2019). Sclerotia in fallen flowers lie dormant on the ground or in plant debris over summer, autumn, and winter. Toward the end of winter, the sclerotia begin to germinate, producing apothecia from which ascospores are released. The pathogen infects and colonizes only flowers, where brown spots appear and spread to the entire organ. Flowers tend to fall prematurely. This causes important damage to the camellia floriculture industry (Taylor and Long, 2000; Denton-Giles et al., 2013).

Studies on the diversity within the genus *Ciborinia* are scarce (Kohn, 1979). The *C. camelliae* variability was investigated using the UP-PCR analysis on strains from America and New Zealand, demonstrating a relatively low level of genetic variations within the two populations (van Toor et al., 2005). On the other hand, a recent study based on morpho-cultural characterization and ITS analysis of numerous Italian strains showed significant variability among the isolates (Saracchi et al., 2022).

The mitochondria are membrane-enclosed compartments that play a central role in providing energy to eukaryotic cells by oxidative phosphorylation (Newmeyer and Ferguson-Miller, 2003; Kriváková et al., 2005; Lv et al., 2017). They are also involved in the signal amplification leading to apoptosis (Goodsell, 2010), antifungal drug resistance as well as in virulence and pathogenicity (Shingu-Vazquez and Traven, 2011; Sandor et al., 2018; Kulik et al., 2020). The study of mitochondrial (mt) genomes reveals interesting features about the pathogen evolution and its relationships with the other related species (Ballard and Whitlock, 2004; Aguileta et al., 2014; Mardanov et al., 2014; Kulik et al., 2020). Even across distantly connected species, the mt genes are largely conserved due to their essential role in cell vitality (Medina et al., 2020). Nevertheless, intron numbers and secondary structures are highly variable and are prone to evolving rapidly. All these features make mitogenome a functional source for phylogenetic studies (Hamari et al., 2002; Burger et al., 2003; Galtier et al., 2009). The mitochondrial genome is often a circular double-stranded molecule with a condensed gene arrangement (Burger et al., 2003). Complete fungal mitogenomes vary notably in size. Typically, the fungal mitochondrial genome contains 14 conserved protein-coding genes involved in the oxidative metabolism (*atp6, atp8, atp9, cob, cox1, cox2, cox3, nad1, nad2, nad3, nad4, nad4L, nad5*, and *nad6*) and one ribosomal protein S3 gene (*rps3*). Additionally, each mtDNA has two ribosomal RNA (rRNA) genes for the large and small rRNA subunit (*rnl* and *rns*) and a variable number of tRNAs (Bullerwell and Lang, 2005).

In this study, we present a comprehensive analysis of the first *C. camelliae* mitochondrial DNA. The complete sequence of an Italian strain was obtained with a hybrid strategy combining Illumina Hiseq paired reads and MinIon Nanopore long reads sequencing. The mitochondrial sequences were identified and assembled together constructing the complete and circular mitogenome. The results were compared with data from the available Sclerotiniaceae mitogenomes. This study allows to position the *C. camelliae* pathogen in the Sclerotiniaceae family and offers some insight into the specificities of mtDNA of *C. camelliae*.

## Materials and Methods

### Sampling, DNA Extraction, and Sequencing

The investigated strain was isolated from pieces of sclerotium collected in Oggebbio (Verbania, Italy) (45°59′47.5782″ N, 08°39′05.9659″ E) and showing *C. camelliae* characteristics. The pathogen was grown on Potato Dextrose Agar medium (PDA: 800 mL/L of potato extract; 20 g/L glucose, BioFROXX, Germany; 15 g/L agar, Applichem, Germany). The isolate was identified according to colony morphology and microscopic analysis of conidia and ascospore traits, as well as ITS sequence (Saracchi et al., 2022). The strain ITAC2 is maintained in the laboratory of Plant Pathology at the Department of Food, Environmental and Nutritional Sciences (University of Milan, Italy), and it is also deposited in a public collection (DSMZ-German Collection of Microorganisms and Cell Cultures GmbH) with the accession number DSM 112729.

High-molecular-weight genomic DNA was extracted from conidia (10^9^ conidia/mL) using DNeasy PowerSoil Pro Kit (Qiagen, Hilden, Germany). DNA was quantified using the Qbit Fluorometer (Invitrogen, Thermo Fisher Scientific—USA) with the Qbit® dsDNA HS Assay Kit (Invitrogen, Thermo Fisher Scientific—USA). The genome quality was checked by spectrophotometer and 1% agarose gel electrophoresis. The gel was visualized using UV-transilluminator Gel Doc 2000 (BIO-RAD laboratories, USA) and Quantity One software (BIO-RAD laboratories, USA) to verify possible smearing of the DNA band.

Two different platforms were used to sequence the genomic DNA: Oxford Nanopore Technologies (ONT) and Illumina Hiseq, executed by Eurofins Genomics (Ebersberg, Germany). The ONT sequencing was performed by the MinION system (FLO-MIN-106 R9.4 flow-cell) using EXP-NBD104, EXPNBD114 in conjunction with the SQK-LSK109 kit and also SQK-RAD004 sequencing kit. A complete mitochondrial genome was obtained by combining long Nanopore reads and short Illumina reads. Sequencing data were analyzed using the European Galaxy web platform (https://usegalaxy.eu/) tools (Afgan et al., 2018) and Geneious Prime software, version 2021.1.1 (Biomatters, Auckland, New Zealand).

The whole genome assembly was performed using Flye v.2.8.3+galaxy0 (Lin et al., 2016) with a default setting. Medaka tool v.1.3.2 +galaxy0 was employed for autopolishing, and mapping of short reads on the draft assembly was performed by minimap2 on the same platform (Li, 2018). The final assembly was obtained using Pilon v.1.20.1 (Walker et al., 2014).

### Mitogenome Assembly

Contigs including the mitochondrial DNA were identified within the whole genome assembly using NCBI data as reference, and BLAST or minimap2 (Li, 2018) as a tool. The detected sequences were extracted and assembled using Geneious Prime software. Single uncalled bases (Ns) and small gaps, resulting from the genome assembly, were fixed by mapping raw reads on the draft assembly and performing manual corrections. Ns and gaps were replaced with the matching bases from illumina reads. Re-mapping was performed to validate the corrections. The mitogenome was circularized by searching the terminal sequences using the minimap2 tool.

### PCR Analysis

After the assembly, the mitochondrial DNA exhibited some unsure repeated regions and sets of uncalled bases (Ns). These mismatches were solved by PCR analysis, using newly designed primers. Primer3web (Untergasser et al., 2012) program was used to design and validate our primers. The new primer pairs were synthesized by Eurofins Genomics (Srl Vimodrone, Italy) (Table 1). The primer pairs 1–2 and 6–7 were used to validate the same sequence amplifying regions with different lengths. The Primer3 was paired with both Primer4 and Primer5. Two PCR rounds were conducted. The first round, using primers 1–2, was performed with 32 cycles in a total 25 μL reaction volume containing: 12.5 μL of Q5^®^ Hot Start High-Fidelity 2X Master Mix (Biolabs, New England), 0.5 μM of forward and reverse primer, 1 μL of DNA sample and the remaining volume of sterile distilled water. The PCR program involved the initial denaturation at 98°C for 2′, 32 cycles of denaturation at 98°C for 20″, annealing at 66°C for 20″, extension at 72°C for 2.5′, followed by the final extension at 72°C for 7′. The second PCR round, using the primer pairs 3–4, 3–5 and 6–7, was performed in a total volume of 30 μL which contained: 0.18 μL of GoTaq® DNA Polymerase 5 U/μL (Promega, Madison, WI, USA), 6 μL of GoTaq® Reaction Buffer 5X (Promega, Madison, WI, USA), 1.2 μL of 10 mM dNTP (Promega, Madison, WI, USA), 1.5 μL of 50 μM forward primer, 1.5 μL of 50 μM reverse primer, 18.62 μL of sterile distilled water and 1 μL of DNA sample. The PCR reaction was carried out with the following parameters: 94°C for 2′; 32 cycles at 95°C for 30″, 55°C for 20″ and 72°C for 45″; 5′ at 72°C. Thermal Cyclers (iCycler-BIO-RAD laboratories, USA and VeritiPro^TM^96-Well Thermal Cycler, Applied Biosystems by Thermo Fisher Scientific) were used to amplify the DNA regions. The reaction products were visualized by electrophoresis on a 1% agarose gel containing ethidium bromide. All PCR products were sequenced using the Sanger technology with the same primers (Eurofins Genomics, Ebersberg, Germany). The sequencing data were analyzed by Geneious Prime software. These sequences were compared to the draft of mitochondrial DNA.

**Table 1:** Primer sequences table.

**Primer name**		**Primer Sequence 5^′^→3^′^**
Primer1	Forward	TCGCGATCCATTACCATCTCT
Primer2	Reverse	CCTAAATTTCACGTGGCATGC
Primer3	Forward	AGTTGGTTGCAGTCGTTTGG
Primer4	Reverse	CAGTTTGGCACCTCGATGTC
Primer5	Reverse	TGACGGGTTTTAATCAGGGGT
Primer6	Forward	AAATGTTCCCTCTGCGTCAG
Primer7	Reverse	TCTACTAAGCGAATAGGTCCACA

### Mitogenome Annotation and Characterization

The mitochondrial genes were annotated using MFannot (http://megasun.bch.umontreal.ca/cgi-bin/mfannot/mfannotInterface.pl) (Beck and Lang, 2010) and RNAweasel (https://megasun.bch.umontreal.ca/cgi-bin/RNAweasel/RNAweaselInterface.pl) (Lang et al., 2007) tools and MITOS WebServer (http://mitos.bioinf.uni-leipzig.de/index.py) (Bernt et al., 2013). Manual corrections were required. To verify the annotation results, sequences and encoded proteins were compared with related species using BLASTp searches against the NCBI database. For each open reading frame (ORF), the protein with the highest similarity was found among biological sequences in the NCBI database. These results were further verified by BLASTn analysis.

### Comparative Analysis

The mitogenome features were compared among some closely related species in the Sclerotiniaceae family, order Helotiales (*Botryotinia fuckeliana* (KC 832409), *Ciboria shiraiana* (CM 017871.1), *C. camelliae* (GCA_001247705.1), *Monilinia fructicola* (NC_056195.1), *Monilinia laxa* (NC_051483.1), *Monilinia polystroma* (GCA_002909645.1), *Sclerotinia borealis* (KJ434027) and *Sclerotinia sclerotiorum* (KT283062). *Glarea lozoyensis* (Order Helotiales; Family Helotiaceae) was used as an outgroup. For all investigated species, data were downloaded from the NCBI database. In species with no mitochondrial genome available, we used BLAST searches to identify the mtDNA in the total genome assembly and subsequently, each detected mitochondrial sequence was annotated according to the procedure described earlier. When possible, these additional results were used in comparative analyses. Based on data availability, seven Sclerotiniaceae species (*Botryotinia fuckeliana, Ciboria shiraiana, C. camelliae* strain ITAC2*, M. fructicola, M. laxa, S. borealis*, and *S. sclerotiorum*) were investigated in terms of gene arrangement. *Monilinia polystroma* and *C. camelliae* ICMP 19812 strain were excluded from this analysis due to the lack of the whole mitochondrial sequence. Multiple mitogenome alignment was performed within the previously cited Sclerotiniaceae using the MAUVE tool of Geneious Prime software and setting *nad4L* gene as starting point. MAUVE alignment identifies homologous regions shared by two or more mitogenomes. These regions are denominated locally collinear blocks (LCBs) (Darling et al., 2004). Tandem Repeat Finder was employed to identify tandem repeats within the mitochondrial genomes (Benson, 1999). The codon usage was estimated using both Sequence Manipulation Suite webserver (Stothard, 2000) and cusp tool of the European Galaxy web platform (https://galaxy-iuc.github.io/emboss-5.0-docs/cusp.html).

The phylogenetic study was performed on the multiple alignments of 14 concatenated mitochondrial proteins using amino acid sequences obtained from each mtDNA. A maximum-likelihood phylogenetic tree was executed by IQ-TREE (Trifinopoulos et al., 2016) web server selecting default setting, and performed by MEGAX software (Kumar et al., 2018).

## Results

The circular mitochondrial genome of *C. camelliae* strain ITAC2, submitted to the GenBank database with the accession number OK326902, has a total length of 114,660 bp. The nucleotide composition is the following: 34.7% of A, 13.2% of C, 16.5% of G, 35.7% of T with a GC content of 29.6%.

**Figure 1:**
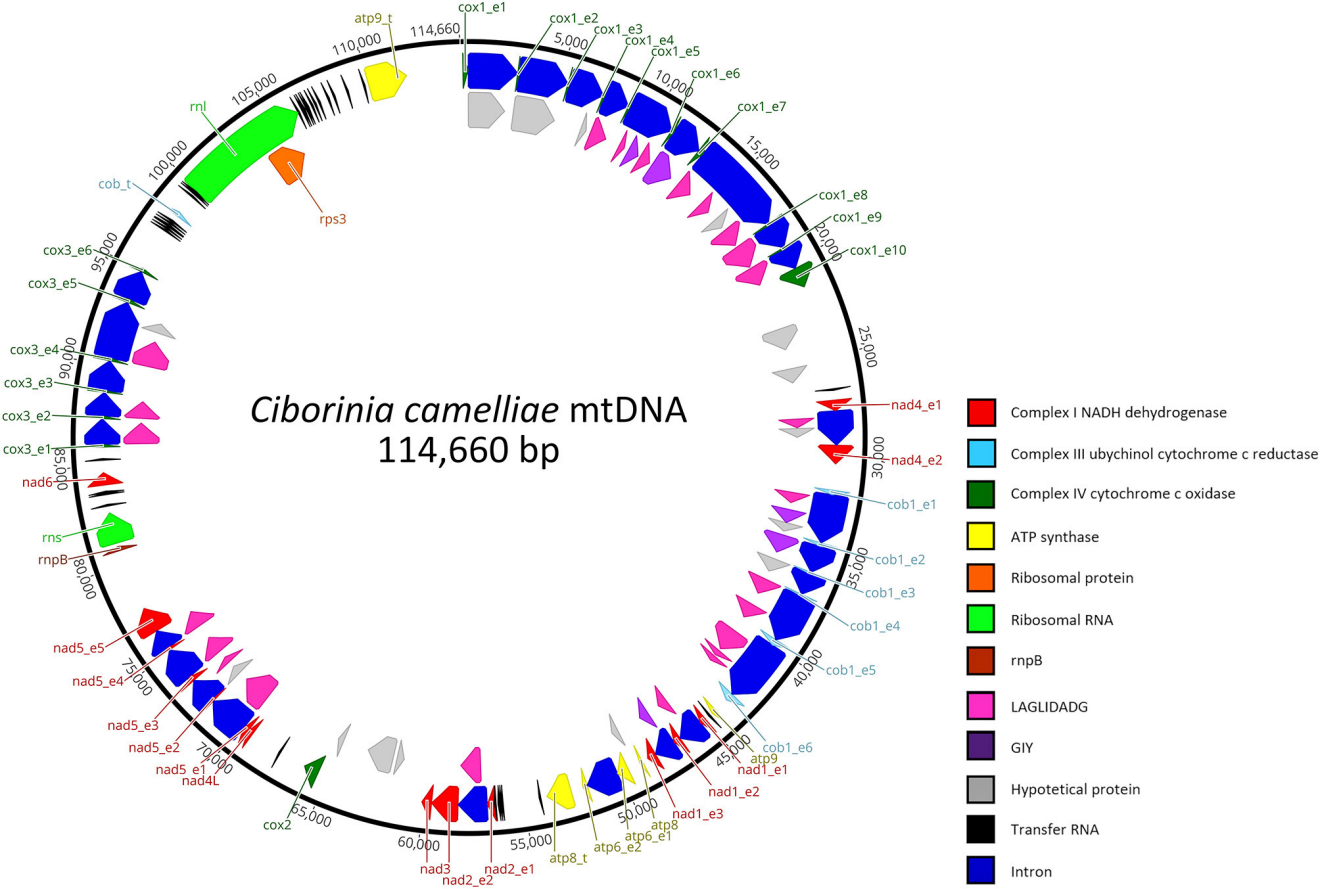
Genetic organization of *Ciborinia camelliae* mitogenome. Circular map is generated by Geneious Prime software, version 2021.1.1 (Biomatters, Auckland, New Zealand). Genes are represented by different colored blocks.

This mitogenome contains all 14 typical genes encoding the subunits of ATP synthase (*atp6, atp8*, and *atp9*), NADH dehydrogenase (*nad1, nad2, nad3, nad4, nad4L, nad5*, and *nad6*), apocytochrome b (*cob*), and cytochrome c oxidase (*cox1, cox2*, and *cox3)*. Additionally, the conserved ribosomal protein-coding gene S3 (*rps3*), untranslated genes of the large and small ribosomal RNAs (*rnl* and *rns*), the ribonuclease P RNA (*rnpB*) gene, and 33 transfer RNAs (tRNA) genes were detected. Overall, the RNA region accounted for 9.26% of the whole mitochondrial genome. The length of individual tRNAs ranged from 69 to 85 bp and most of these genes were placed around the *rnl* region (Figure 1). The total tRNAs are related to 18 essential amino acids.

In total, the mitogenome contains 62 putative genes, including 44 open reading frames (ORFs) for hypothetical proteins (15) and homing endonucleases of the LAGLIDADG (24) and GIY-YIG (5) families. BLASTp analysis was conducted for these non-conserved ORFs. The results (Supplementary Data Sheet 1), also verified by BLASTn analysis indicate that 22 ORFs have high identities with mitochondrial ORFs of the genus *Monilinia*. On the other hand, a high similarity with the Sclerotiniaceae species was not observed in 14 % of the ORFs. These exhibit the highest amino acid identity (ranging from 63.41 to 90.75%) with fungal species belonging to the family of Nectriaceae, Ceratocystidaceae, Cryphonectriaceae, Erysiphaceae, and Orbiliaceae. The last two are respectively evolutionary the closest and the most distant from the Sclerotiniaceae family. A LAGLIDADG of *Golovinomyces cichoracearum* and one of *Fusarium tricinctum* are the most representative ORFs with the highest amino acid identity: 85 and 90.75% with 100 and 92 % of coverage, respectively.

The coding sequences (CDS) of the 14 core mitochondrial proteins and *rps3* were used in the codon usage analysis. This investigation was carried out among the selected Sclerotiniaceae species and *F. oxysporum* as external control (Supplementary Data Sheet 2). The results did not show significant variability in the use of the codons. All the investigated species showed the same preferential codons, encoding the same amino acid, except for Valine and Threonine (Table 2). The most used codons for Threonine are ACA and ACT. In *C. camelliae* strain ITAC2, *S. sclerotiorum, M. laxa, C. shiraiana*, and *B. fuckeliana* the preferential codon is ACA, while in *M. fructicola, S. borealis*, and *G. lozoyensis* is ACT. The preferential codon for Valine is GTT for all investigated species, except *Monilia fructicola*, which uses more frequently GTA. Nevertheless, the GTA codon represents the second choice to encode Valine also in the other analyzed species.

**Table 2:** Codon usage patterns for the 14 core mitochondrial protein coding-genes and *rps3* in Sclerotiniaceae species (*Ciborinia camelliae* strain ITAC2, *Botryotinia fuckeliana, Ciboria shiraiana, Monilinia fructicola, Monilinia laxa, Sclerotinia borealis, Sclerotinia sclerotiorum*) and *Glarea lozoyensis*.

	**Preferential codon**							
**Amino acid**	***Ciborinia camelliae*** **ITAC2**	***Botryotinia fuckeliana***	***Ciboria shiraiana***	***Monilinia fructicola***	***Monilinia laxa***	***Sclerotinia borealis***	***Sclerotinia sclerotiorum***	***Glarea lozoyensis***
Ala	GCT	GCT	GCT	GCT	GCT	GCT	GCT	GCT
Cys	TGT	TGT	TGT	TGT	TGT	TGT	TGT	TGT
Asp	GAT	GAT	GAT	GAT	GAT	GAT	GAT	GAT
Glu	GAA	GAA	GAA	GAA	GAA	GAA	GAA	GAA
Phe	TTT	TTT	TTT	TTT	TTT	TTT	TTT	TTT
Gly	GGT	GGT	GGT	GGT	GGT	GGT	GGT	GGT
His	CAT	CAT	CAT	CAT	CAT	CAT	CAT	CAT
Ile	ATA	ATA	ATA	ATA	ATA	ATA	ATA	ATA
Lys	AAA	AAA	AAA	AAA	AAA	AAA	AAA	AAA
Leu	TTA	TTA	TTA	TTA	TTA	TTA	TTA	TTA
Met	ATG	ATG	ATG	ATG	ATG	ATG	ATG	ATG
Asn	AAT	AAT	AAT	AAT	AAT	AAT	AAT	AAT
Pro	CCT	CCT	CCT	CCT	CCT	CCT	CCT	CCT
Gln	CAA	CAA	CAA	CAA	CAA	CAA	CAA	CAA
Arg	AGA	AGA	AGA	AGA	AGA	AGA	AGA	AGA
Ser	AGT	AGT	AGT	AGT	AGT	AGT	AGT	AGT
Thr	ACA	ACA	ACA	ACT	ACA	ACT	ACA	ACT
Val	GGT	GGT	GGT	GGA	GTT	GGT	GGT	GGT
Trp	TGA	TGA	TGA	TGA	TGA	TGA	TGA	TGA
Tyr	TAT	TAT	TAT	TAT	TAT	TAT	TAT	TAT
Stop codon	TAA	TAA	TAA	TAA	TAA	TAA	TAA	TAA

Focusing on gene-specific codon usage, only in the *nad2* gene do all the investigated Sclerotiniaceae use the same preferential codons, suggesting that the *nad2* is the most conserved gene, followed by the *atp8* and *rps3* genes showing differences in only two amino acids. On the other hand, *cox1, atp9, nad3, cob*, and *nad4L* are the genes with the greatest differences in codons usage. They exhibited dissimilarity from 9 to 12 out of 21 amino acids (Supplementary Data Sheet 3).

Only for the *C. camelliae* ITAC2 strain, the codon usage of non-conserved ORFs was investigated and compared with that of conserved genes. The ORFs and the conserved regions exhibited a similar codon usage, excluding Isoleucine (ATA in conserved genes, ATT in ORFs) and Threonine amino acids (ACA and ACT in conserved regions and ORFs, respectively) (Supplementary Data Sheet 4).

The mitochondrial genome size is associated with a high content of introns (Deng et al., 2018). In *C. camelliae* mtDNA we detected 28 introns, which are located in eight out of 14 genes: *atp6* (1), *cob* (5), *cox1* (9), *cox3* (5), *nad1* (2), *nad2* (1), *nad4* (1) and *nad5* (4) (Table 3). Introns exhibit a variable length ranging from 1,015 bp to 4,750 bp. The *cox3, cox1*, and *cob* genes showed the highest fraction of intronic sequences: 91.2, 90.6, and 90.1%, respectively. For each gene containing introns, the exon protein-coding sequence (CDS) was <54%. In the 14 mitochondrial genes, the portion of non-coding DNA ranged from 0 to 68% (Table 3). The total content of non-coding DNA in the ITAC2 strain mitogenome is ~48%. The sequences encoding the fourteen core mitochondrial proteins and *rps3* protein represent only 13.3% of the entire mitogenome.

**Table 3:** List of the 15 mt genes with their: total length, introns, protein coding sequence (CDS) size and the portion of non-coding DNA.

**Gene**	**Gene size (bp)**	**n°of introns**	**Intronic size %**	**Exon CDS size (bp)**	**Intron CDS size (bp)**	**% non-coding DNA**
*atp6*	2,436	1	68	777	0	68
*atp8*	144	0	0	144	0	0
*atp9*	222	0	0	222	0	0
*cob*	11,868	5	90.1	1,173	7,134	30
*cox1*	21,255	9	90.6	1,995	14,215	23.7
*cox2*	753	0	0	753	0	0
*cox3*	9,176	5	91.2	807	4,077	46.8
*nad1*	3,488	2	68.8	1,089	1,062	38.3
*nad2*	3,167	1	46.5	1,695	1,083	12.3
*nad3*	459	0	0	459	0	0
*nad4*	3,164	1	53.6	1,467	948	22.6
*nad4L*	267	0	0	267	0	0
*nad5*	8,052	4	75.4	1,983	4,251	18.5
*nad6*	672	0	0	672	0	0
*rps3*	1,743	0	0	1,743	0	0

*Intron CDS size is the length of sequences encoding ORFs*.

The mtDNA of *C. camelliae* contains incomplete duplicated copies of *atp8, atp9*, and *cob* genes. All of these extra copies appeared truncated. The *atp8*-like ORF is located between *atp6* and *nad2* genes. The first 33 amino acids (aa) exhibit 75 % of similarity with the *atp8* protein. Instead, no significant result was found for the remaining 385 aa. The *atp9* copy is placed between *cox1* and *rnl* region. The first 59 amino acids show 100% identity with the *atp9* protein. This similarity decreases for what concerns the rest of the protein. The *cob*-like ORF was detected between *cox3* and *rnl* region and consists of only 68 aa with an identity of 80% compared to the *cob* gene. All three extra copies were observed also in *C. camelliae* ICMP 19812 strain from New Zealand (Supplementary Data Sheet 5). The extra genes similarity between the two *C. camelliae* strains ranges from 87.9 to 100%. The variability between the two strains was especially due to the lack of sequences or uncalled bases (Ns) in the New Zealand whole genome assembly.

A total of 36 tandem repeats were found within the *C. camelliae* mitogenome. The two longest repeat sequences measure 218 and 101 bp, respectively. The first is located in an intergenic region among tRNA genes. The second is located in the third intron of *cox1*. In total, the pair-wise nucleotide similarities ranged from 71 to 100%. Many of these regions were repeated approximately twice. The highest repetition was 7. Repetitive sequences were found also in the other investigated species (Table 4).

**Table 4:** Comparison of *Ciborinia camelliae* strain ITAC2 mitogenome (OK326902) with closely related species: *Botryotinia fuckeliana* (KC 832409), *Ciboria shiraiana* (CM 017871.1), *Monilinia fructicola* (NC_056195.1), *Monilinia laxa* (NC_051483.1), *Sclerotinia borealis* (KJ434027), *Sclerotinia sclerotiorum* (KT283062) and *Glarea lozoyensis* (NC_031375.1) as outgroup.

**Genomes features**	***Ciborinia camelliae*** **ITAC2**	***Botryotinia fuckeliana***	***Ciboria shiraiana***	***Monilinia fructicola***	***Monilinia laxa***	***Sclerotinia borealis***	***Sclerotinia sclerotiorum***	***Glarea lozoyensis***
Mitogenome size (bp)	114,660	82,212	156,608	159,648	178,357	203,051	128,852	45,501
GC content (%)	29.6	29.9	30.9	30.9	30.1	32.9	30.9	29.8
n° of conserved protein-coding genes	15	15	15	15	15	15	15	15
rRNA	2	2	2	2	2	2	2	2
tRNA	33	31	36	32	32	31	33	34
n° of introns	28	20	28	35	33	52	23	4
n° of non-conserved ORFs	44	22	77	51	94	76	30	6
Tandem repeats	36	20	27	36	60	67	27	12
n°of gene copies	3	1	1	1	0	1	1	1

Pearson's correlation analysis was performed in species reported in Table 4. The association between the mitogenome size and the number of introns, non-conserved ORFs and tandem repeats was supported by statistical data (Supplementary Data Sheet 6). Each independent variable exhibited a high correlation coefficient with the mitogenome size, ranging from 0.929 to 0.869 (*p*-value < 0.05).

**Figure 2:**
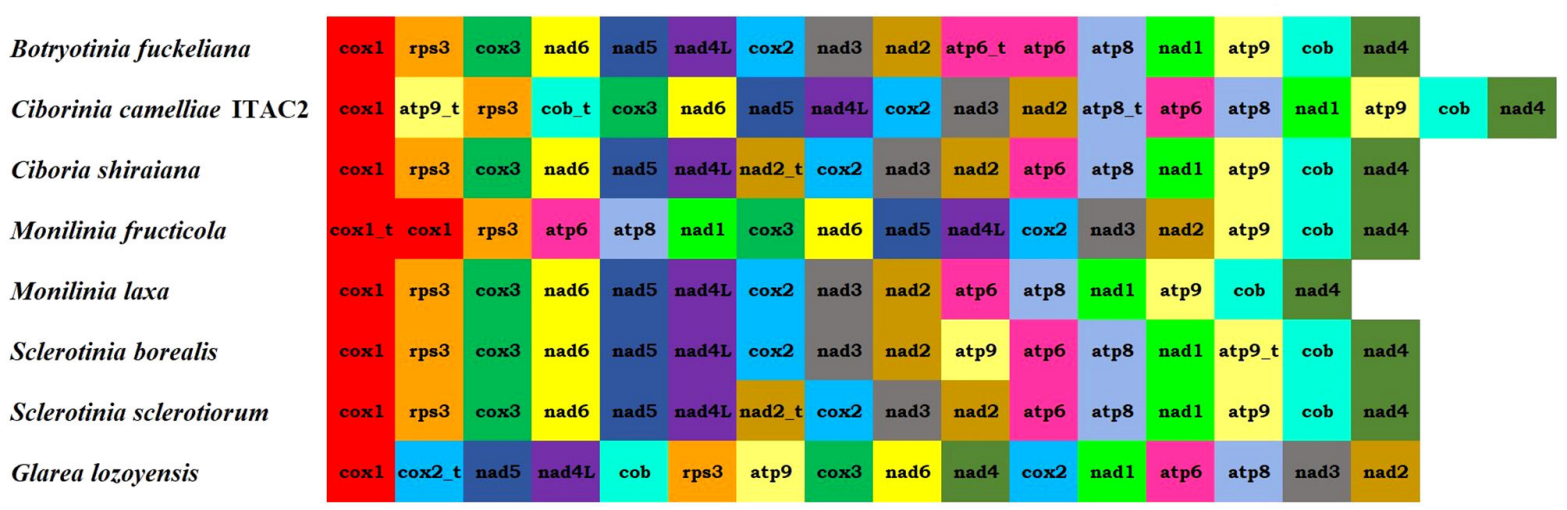
Mitogenome organization of the seven Sclerotiniaceae investigated [*Botryotinia fuckeliana* (KC 832409)*, Ciboria shiraiana* (CM 017871.1)*, Ciborinia camelliae* strain ITAC2 (OK326902)*, Monilinia fructicola* (NC_056195.1), *Monilinia laxa* (NC_051483.1)*, Sclerotinia borealis* (KJ434027) and *Sclerotinia sclerotiorum* (KT283062)] and *Glarea lozoyensis* (NC_031375.1) as outgroup.

The arrangements of the 15 protein-coding genes were compared among some related species (Figure 2). Gene order in *C. camelliae* is identical to most of the Sclerotiniaceae species, except for minor differences due to replication events of *atp8, atp9*, and *cob* genes. Among Sclerotiniaceae, gene arrangement in *M. fructicola* appears with more evident differences. Nevertheless, all investigated species preserved four synteny units: *nad5*-*nad4L, nad3*-*nad2, atp6*-*atp8*, and *cox3*-*nad6*. The location of *atp9* and *cob* truncated genes of *C. camelliae* could be connected to the gene order of *Glarea lozoyensis*, even if reversed. The position of the *atp8* truncated gene between *nad2* and *atp6* was found also in *Aspergillus flavus* (NC_026920.1).

**Figure 3:**
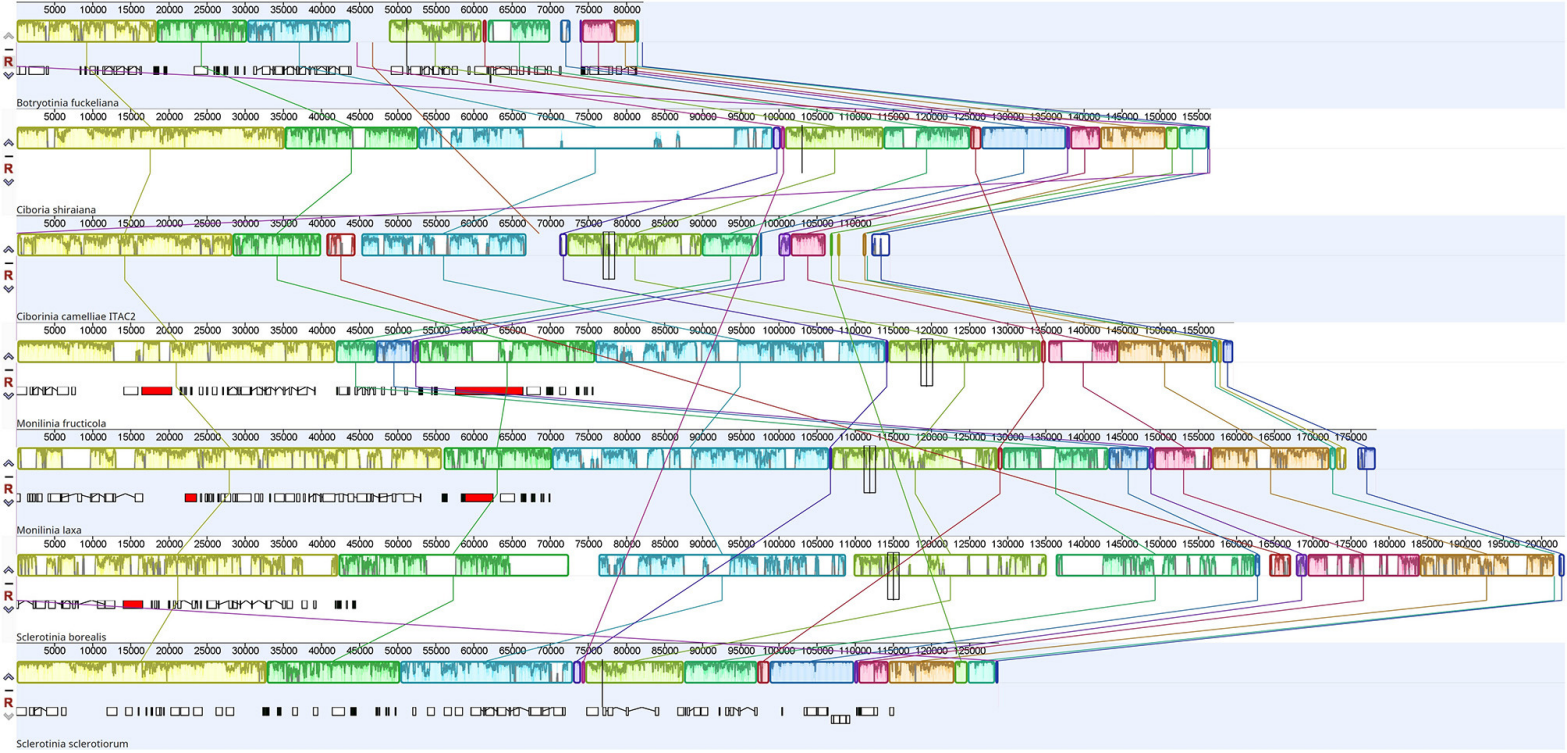
Mauve alignment of Sclerotiniaceae mitochondrial genomes (*Botryotinia fuckeliana, Ciboria shiraiana, Ciborinia camelliae* strain ITAC2, *Monilinia fructicola, Monilinia laxa, Sclerotinia borealis* and *Sclerotinia sclerotiorum*). The alignment was performed setting *nad4L* gene as origin. The colored blocks reveal homologous regions between mitogenomes. Each block exhibits nucleotide similarity profile. Homologous sites are connected by lines.

The Mauve alignment was performed only considering Sclerotiniaceae species. This investigation revealed the presence of 19 homologous regions between the seven species (Figure 3). The length of homologous sites ranged from 128 bp to 115,310 bp. The two largest regions contain respectively the *cox1* gene and *nad4L, nad5, rns, nad6*, and *cox3* gene. A homologous region (5 Kbp), containing the truncated *atp9* gene, was found only in *C. camelliae* and *S. borealis* mitogenomes.

**Figure 4:**
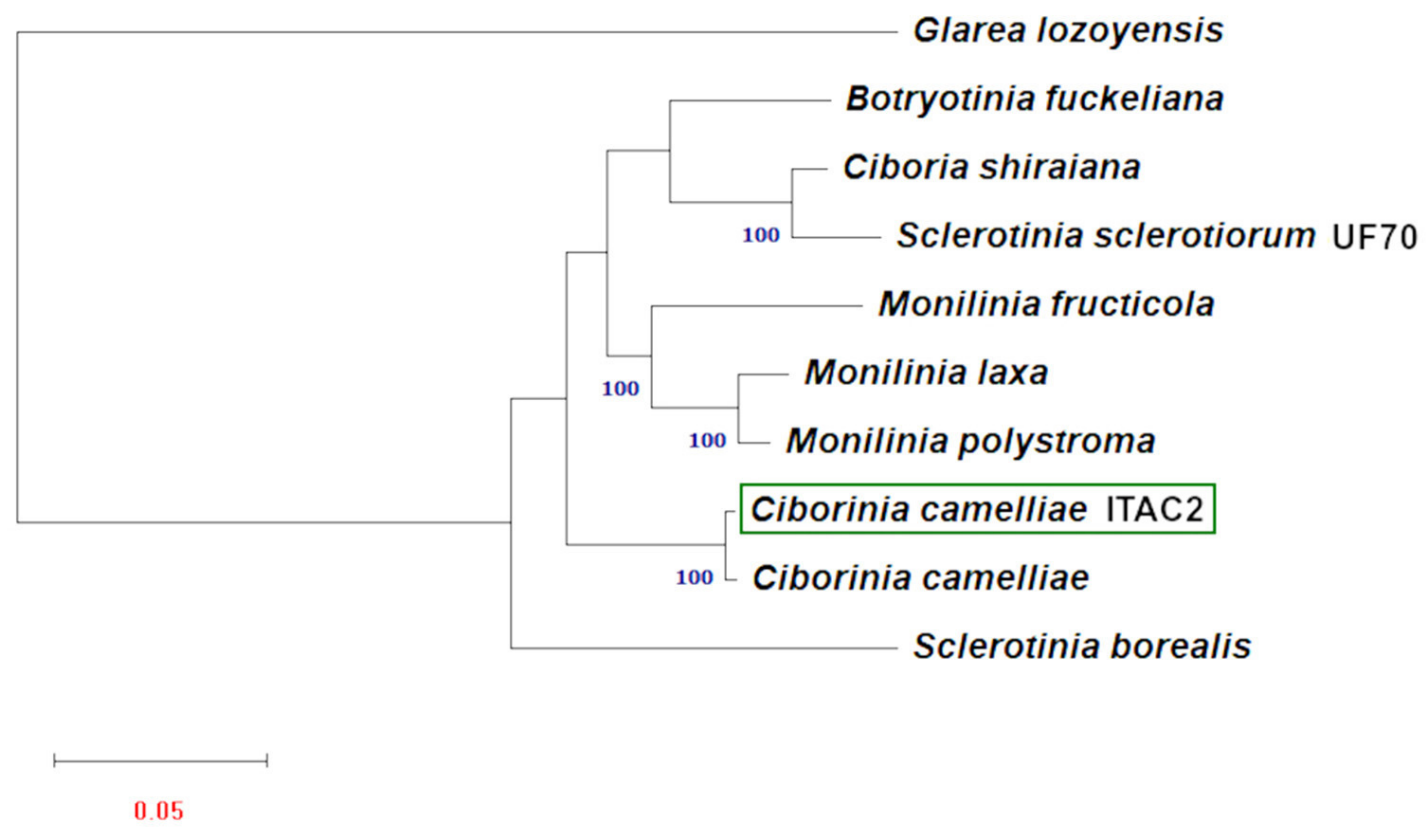
Maximum Likelihood phylogenetic tree based on 14 encoded mitochondrial genome. Blue numbers are bootstrap support values (>90). Red number denotes branch length. The GeneBank accession numbers are listed as follow: *Botryotinia fuckeliana* (KC 832409), *Ciboria shiraiana* (CM 017871.1), *Ciborinia camelliae* (GCA_001247705.1), *Ciborinia camelliae* strain ITAC2 (OK326902) *Glarea lozoyensis* (NC_031375.1), *Monilinia fructicola* (NC_056195.1), *Monilinia laxa* (NC_051483.1), *Monilinia polystroma* (GCA_002909645.1), *Sclerotinia borealis* (KJ434027) and *Sclerotinia sclerotiorum* (KT283062).

Phylogenetic analysis was carried out using the amino acid sequences of 14 protein-coding mitochondrial genes. Nine Sclerotiniaceae species with publicly available data were investigated. To obtain a second mitochondrial set of genes from another *C. camelliae*, a partial mitochondrial genome composed of a contig list was obtained from the whole genome assembly of *C. camelliae* ICMP 19812 strain from New Zealand (Supplementary Data Sheet 5). *Glarea lozoyensis* was considered as an outgroup. The phylogenetic tree (Figure 4), executed according to the model VT+F+R3, demonstrated a close connection between *C. camelliae* ITAC2 strain and the members of the Sclerotiniaceae family. The two *C. camelliae* strains group as an independent clade, distinct from the other Sclerotiniaceae species. They exhibit 95.4 and 98.3% of similarity in nucleotide and amino acid sequence, respectively.

## Discussion

Nowadays, the growth of high-throughput sequencing technologies has improved phylogenetic works using mitogenomes (Zardoya, 2020). In this study, the first mitochondrial genome of *C. camelliae* was described demonstrating the phylogenetic position of the species in the Sclerotiniaceae family. The mitochondrial genome of *C. camelliae* was obtained by combining Illumina and Nanopore reads, complemented with Sanger sequencing of specific PCR products to verify the assembly. Experimentally, the combination of long and short reads allowed us to obtain a good quality mitochondrial genome as observed for other species (Degradi et al., 2021). Employing different sequencing technologies was useful to verify the existence of repetitive elements and to define their length and position in the mitogenome assembly (Kinkar et al., 2021). The Sclerotiniaceae mitogenomes were already investigated previously (Mardanov et al., 2014; Yildiz and Ozkilinc, 2020, 2021). Our work contributes to improving the knowledge on mitogenomes of the Sclerotiniaceae family by performing comparative analysis. According to the NCBI data, the *C. camelliae* strain ITAC2 mitogenome with its 114,660 bp is confirmed in the range from 82 to 203 kbp of the Sclerotiniaceae species (Table 4). Within this family, *B. fuckeliana* and *C. camelliae* exhibit the most compact mitochondrial genomes. Among the investigated species, *C. camelliae* mitogenome contains the highest number of truncated gene copies (*atp8, atp9*, and *cob*). Intermediate values in the number of non-conserved open reading frames (ORFs), introns, and tandem repeats were detected. According to Pearson's correlation analysis, the distribution of these elements, due to the evolutionary process, contributes to the high variability in mitogenome size among closely related species (Jung et al., 2010; Xiao et al., 2017; Chen et al., 2021). The intronic regions resulted the greatest promoters of variations in mitogenomes size (Li Q. et al., 2021).

Excluding the events of gene duplication, the *C. camelliae* gene arrangement is identical to most of the Sclerotiniaceae. All the examined species exhibited four syntenic units: *nad5-nad4L, nad3-nad2, atp6-atp8*, and *cox3-nad6*. These clusters were preserved also in the MAUVE alignment. One of the largest LCBs contains the *nad4L-nad5* and *nad6-cox3* pairs. The homologous regions measuring 22 and 38 Kbp include the *nad2-nad3* and *atp6-atp8* genes. These conserved clusters could have originated from a common ancestral gene order and later assumed a different organization. Some Helotiales species, such as *Phialocephala subalpine, Rhynchosporium orthosporum*, or *Rhynchosporium secalis*, evolutionary close to Sclerotiniaceae, exhibit the unit *nad4-nad1*, also found in some Sordariomycetes and Eurotiomycetes (Pantou et al., 2006). This cluster was instead split by *atp9* and *cob* genes in Sclerotiniaceae. In all investigated species, the *rps3* gene is located in the *rnl* region and is not interrupted by introns.

Codon usage analysis showed similar use of the optimal codons among the related species (*C. camelliae, B. fuckeliana, C. shiraiana, M. fructicola, M. laxa, S. borealis*, and *S. sclerotiorum*). The main differences were observed in the use of codons encoding Valine and Threonine amino acids. The same results were obtained considering less related species such as *Glarea lozoyensis* (NC_031375.1) and *Fusarium oxysporum* (NC_017930.1), suggesting the preferential use of codons is conserved among more evolutionarily distant species of fungi. Our study indicates the presence of more conserved mitochondrial regions (*nad2, atp8*, and *rps3*) and others prone to more variability (*cox1, atp9, nad3, cob*, and *nad4L*). For example, all the investigated Sclerotiniaceae showed the same codon usage in the *nad2* gene, while *atp9* and *nad3* exhibited dissimilarities in 12 amino acids. This variability may be due to weak natural selection toward synonymous substitutions not resulting in amino acid modifications (Stewart et al., 2011). Also the ORFs and the conserved regions of *C. camelliae* mtDNA showed a similar codon usage suggesting the same selective pressure for translational efficiency (Whittle et al., 2012). Nevertheless, some differences between conserved and non-conserved ORFs were observed in the differential use of synonymous codons, namely codon usage bias (CUB). The codon bias has been reported in many organisms (Duret, 2002; Sharp et al., 2005; Xiang et al., 2015; Gupta and Singh, 2021) including mitochondrial genomes (Wei et al., 2014; Yildiz and Ozkilinc, 2021). Highly-expressed genes exhibit a strong bias to conserve their translational efficiency. On the other hand, CUB decreases in non-conserved genes due to weak selection, thus they can afford to use different codons having low expression (Bulmer, 1991). On the contrary, a low CUB in weakly expressed ORFs is a mechanism for preserving a low level of expression (Grosjean and Fiers, 1982). The influence of selective pressure on the codon bias makes CUB an interesting source of species evolution force leading to their environmental adaptation (Angellotti et al., 2007; Li X. et al., 2021), which include host-pathogen relationships (Gupta and Singh, 2021). Further studies may investigate the functional role of CUB in fungal mitochondrial genomes.

As observed in other fungi (Mardanov et al., 2014; Yildiz and Ozkilinc, 2021), only the Methionine amino acid ends in G, instead, the last base of all the other preferential codons is A or T confirming the high AT content of fungal mitogenomes (Chen et al., 2019; Zhang et al., 2020).

The BLASTp analysis of the non-conserved ORFs was useful for revealing phylogenetic relationships among species. All ORFs exhibiting a significant hit in BLAST analysis, showed the best similarity with an Ascomycota species. Sixty-six percent of ORFs demonstrated the highest similarity with ORFs from the Sclerotiniaceae species. According to BLAST results, six of *C. camelliae* ORFs could have origins distinct from the Sclerotiniaceae family. Two interesting LAGLIDADG, located in *nad5* and *cox1* introns, showed 90.75 and 85% of amino acid identity with *F. tricinctum* and *G. cichoracearum*, respectively. These regions may represent events of horizontal transmission from distant fungal species, given the ability of intron I and homing endonuclease genes to move and integrate into diverse genomes (Beaudet et al., 2013; Celis et al., 2017).

The phylogenetic study based on multiple alignment of 14 concatenated mtDNA-encoded protein-related sequences further confirmed that *C. camelliae* belongs to the family of Sclerotiniaceae and its relationships among strictly related species. Phylogenetic results showed a significant difference between the two *Sclerotinia* species. Our results are partially confirmed by other works on mitochondrial DNA, where the dissimilarity between *S. borealis* and *S. sclerotiorum* was reported (Mardanov et al., 2014; Ma et al., 2019; Yildiz and Ozkilinc, 2021). The nucleotide and amino acid similarity between the two *Sclerotinia* species are 36.4 and 77.7%, respectively. Discrepancies between mitochondrial and nuclear phylogeny shall be further investigated in this genus. This phylogenetic analysis proved the closest match between the strain ITAC2 and the strain ICMP 19812 of *C. camelliae* from New Zealand, with which they constitute an independent monophyletic group. Nevertheless, some differences between the two strains at the nucleotide and amino acid sequence level could be detected in the mitochondrial genome (95.4 and 98.3% of similarity, respectively). The major level contributor to the diversity in numbers is the *cox1* gene. The Italian and New Zealand *cox1* genes exhibit 88.3% of amino acid identity, due to the deletion of some amino acids located at the beginning of *cox1* in the strain from New Zealand. According to BLAST search, the first exon of the Italian strain results lacking in the New Zealand strain. Excluding this gene, the amino acid identity between the two strains would be 99.8%. According to Mardanov et al. (2014), *cox1* is the most common target of intron insertions. This means that *cox1* represents a useful marker in phylogenetic studies, as it is evolving more rapidly than the other mitochondrial genes. The significant difference between the two *C. camelliae* strains may be due to a misassembly of the New Zealand strain genome or could represent the beginning of a speciation event. The Italian and New Zealand population are separated by geographic barriers and they may diverge in complete independence under different selection pressures (Dettman et al., 2008; Stukenbrock, 2013). Moreover, the impact of globalization on the diffusion of the phytopathogens influences the evolutionary process resulting in more variability. One day, the subsequent differentiation of populations may evolve into two different species. Large sequencing of *C. camelliae* population is warranted to further investigate the worldwide diversity of the species.

## Data Availability Statement

The datasets presented in this study can be found in online repositories. The names of the repository/repositories and accession number(s) can be found below: GenBank [accession: OK326902].

## Author Contributions

MP, MS, and PC designed the work. IV, LD, and AK performed the experiments and acquired data. MP, MS, IV, and LD analyzed and interpreted the data for the work. IV, MP, MS, and AK drafted the manuscript. All authors revised critically the manuscript.

## Conflict of Interest

The authors declare that the research was conducted in the absence of any commercial or financial relationships that could be construed as a potential conflict of interest.

## Publisher's Note

All claims expressed in this article are solely those of the authors and do not necessarily represent those of their affiliated organizations, or those of the publisher, the editors and the reviewers. Any product that may be evaluated in this article, or claim that may be made by its manufacturer, is not guaranteed or endorsed by the publisher.
