# Supplementary material for: The First Mitochondrial Genome of Ciborinia camelliae and Its Position in the Sclerotiniaceae Family
Source: Front Fungal Biol. 2022 Feb 9;2:802511. doi: 10.3389/ffunb.2021.802511 (PMC10512376; doi:10.3389/ffunb.2021.802511)
Supplement: Supplementary file 5 [file Data_Sheet_5.docx]

**mtDNA *Ciborinia camelliae* GCA_00124770.5**

The list includes 19 contigs.

Each contig is followed by its annotation.

1. LGKQ01000652.1 (reversed)

>Feature LGKQ01000652.1 (reversed) Table

132 1805 gene

gene orf557

132 1805 CDS

product hypothetical protein

translation: "MERGDCLSVGASPTYGNGASVVGLLPKGGRKWRIESNSQHNGFRCYSKA

VMKTAKGDLPRRFEKLTQMCAAHKDGFKANDIYKLMFNVRMYEVASHKLGSRAGNMTPGRTPVRLDGKSQEWIKETID

QMRDGTFQFKPGRRVLNPKRGSSNTRPLTIAPPRDKVVLEVMRMILEAIFETTFSDNSHGFRPKRNSHTALRQVKTQF

GAVTTYIEGDISKCFDSFDHKILIDLVKRRVSDTRFIQLIWKALRAGYMEFHTSQHSIIDTLQGSIVSPLLANIYLHE

LDLYIEKLKTNYDKGGVASKNPEYRKLEYQRSKANKAGDSTLGKKYLKRMQQTNSRLTNDPKFRRIYYVRYAEYWFMA

VRGPRSDSVDMLQSIRQMLGNSLKLDLSVEKSKIIHPRVEPALFLGTLISISNHNGYTRGNNHQKVKVASQIRMLAPM

DRIFNKLTIGGFMSAKYKSGIPRFIWLANNKDSIIKLYNSVLSGYLDYYRFTHNYPRVASSLEFILKTSCAKLLAAKY

KLGSVTKVIKRYGEDLKGKEKIGFLKPCYKLNVWEFKSKP"

>2271 4622 gene

gene orf783

>2271 4622 CDS

product hypothetical protein, partial

transl_except (pos:2271..2273, aa : A)

translation: "APFNIFFKSGVVVPKGCCHFLLNQLTYLKLKGMRECSMSVKPYLSTVEQ

SEEGGKVEVFYALISTTTVGLCVSVGFSLTQALHQMLVIMSASVTKDNHLVTTSKRGKVVYNAKGERRFGIWNSGLPK

RRNPHGNGSAIVGMIPGIVRSIHTEVRKLDNSPKLETQVGQKSLGEMLMFDTEGKCNNAYRILCESSIIQSAYMRIKS

EPGNMTPGHDKETLDGISEGWFAKTITDLIKEKFTFKPTRRVFIPKANGKMRPLGIGSPRDKIIQEAFRAILEAVLES

KFSDKSHGFRPGRGCHSALAQIRYWNGIKWFIEGDIKAFFDNIDHHILEKLLRKHFNDQRFIDLYWKMVRAGYVEFGN

KSSSVIGVPQGGIASPILSNLILNELDRYIELLLEENNRKLQSKSHTVRNPAYYSLDYRIQGITKLERKWKALGKTLD

LNRKTERLELIKVRSKIPSTIPHPELARFYYVRYADDWLIGVAGPSSFAGELKNKISVFLRDELKLELSQVKTLITNA

AKGKAHFLGTEIYRTSSVKGEIKRFKNTRGHSQIIPTTSIVMNAPISKIVSKFVGKELVTWTSSVLSEDNLNPLPILK

WVNLPIRDIILRYRMILNGVLNYYTFANNKPSLILIYWILRKSLAKTLATKLKLGSARKVYLKFGIDIKYLIPETDKV

IDFARPSLLPTPKKFLGNTDFTDSLRVVEWKLRTVNFFNYVCSSCGSSEDLQVHHLKHIRTIDADLNGFDKQMAAINR

KQIPLCRKCHHKVHTGNYDGMSLKILNKIKTD"

6156 6485 gene

gene orf109

6156 6485 CDS

product hypothetical protein

translation: "MPEYLLEVGPFIVLLIFVIDLTILTIFLATNASRARPEVVEQGKAELGK

QIKELGENRRERNLDAEQYSRERAANDPNAEKTAEDVGQWDELINEGQRTIVDIINWLSS"

>6730 7650 gene

gene orf306

>6730 7650 CDS

product hypothetical protein, partial

note LAGLIDADG

transl_except (pos:6730..6732, aa : K)

translation: "KGFGPHPTGLAGVGKILSTKYSGTSSLPLNSCNLGSYLAGLFEGDGHIW

IQKQVGKKTHNPRFCITFSLKNEALAKKLLGIIGSGFLRYKPKDNACVIVVSPVVGLKRIVDLINGELKTPKINQLHN

LIDWLNKNHSARLNKLPLNKDILENSSWLSGFVDADGSFSVQHTKTETGAAKRKISCRLRIEQRILDPSTHEDYFSIL

NQICLFFNCKLLTRRQKSTGNTYYTLAASSKASLNIIINYFNKYPLFSSKFLDYQDWELVAKLFVNNQHLTPEGINTV

EMVRSRMNTKRVDFNWDHLISLY"

8497 8811 gene

gene orf104

8497 8811 CDS

product hypothetical protein

note LAGLIDADG

translation: "MLGHYLAGLMEGDGSIIVPVTNRNQKGKLLYPKIKITFVDKDAPLAIKL

QEVFGAGTLEYPKNTKYVNLLFQDVNSLWTIAVLLNGKMRTPKIEARGGSTIVII"

9061 9678 gene

gene orf205

9061 9678 CDS

product hypothetical protein

note GIY

translation: “MDSDKKFVVAENRGKPGVYRLINLTNENVYVGSSADLGRRFTSYYSFMY

IDAQKTSLICKALLKYGYSKFRVEVLEYCTLENLLEREQYYIDLLKPKYNILTIAGSSLGYKHTEETIDKFKARRHTK

ETIELFKARTFSEETRAKISAVNGTKVIVCDLFTNNFSEYVSIRKAALGLNAPYSTIRYCTAQNKLYMDRYQITLKNK”

9821 10417 gene

gene orf198

9821 10417 CDS

product hypothetical protein

note LAGLIDADG

translation: "MINWLNARSTSTSKLPFNNLKLTKLELDTSHLGDNPWLTGFIEADGNFY

SNFSVNSQGIAEEIRHYMRISQKAVYGKKSNLFNEDYSNKHIMEKIREFLDVKSVNEIKRTKEEFVELAYEVRTSKKS

SSEKLIAYLSKYPLFSSKHQDFLSWGEIHKIRLFKLYKTLDGTKRLILLKNSMNTLRTQYNWDSLNRFYTI"

>10591 11868 gene

gene orf425

>10591 11868 CDS

product hypothetical protein, partial

note GIY

transl_except (pos:10591..10593, aa : G)

translation: "GFGFFTITFLILFLFLKFSFTANNKSAYNNGRGNLNCEDKSEDNSEDNN

FSGPDPEEPDNNLSDPEPEQSEEPEEPEDPEESDTDDRSSKNDSNKDWGLILGWKGNNIFAHQIAKAQIKSGKPVSIN

VLNKILAYSGILVNEETLDSLINIPRLIFKDLHKDATRELIDETLGLPHSKIQQRGVYIFTITDTNQKYVGSSSQLAL

RLRGYLNQTHKKAGKLIPLIEEKGLACFKLEVICLPYYPDFRPEIVLEQYFLLDPSFSLNTIKVSNNPSGSTAKRLYM

YNRDGSILYYFTTQQKDFISKLNISHFTFTKHLTKGTYYLGKYLFLRERIGTAKVTEMTLPEIAIMLQQDRVNFNKSK

PVNSLSKRVLLIDIQSEEEIVFESLGKCAIFFSSKGFPFSHSTLVKRLDTNIPYRGYICKTQIK"

>12374 13303 gene

gene orf309

>12374 13303 CDS

product hypothetical protein, partial

note LAGLIDADG

transl_except (pos:12374..12376, aa : V)

translation: "VFENYFILLTLFVFPSSNQLIFKRNISKFNSNLNHKFDFSAFYKKYNTH

LPNNKIPSENFLTWLVGFTEGEGSFIVNNRGDLAFVITQTTIDKQVLEFIQEILGFGKVIAQSTITSRYVTQNKKEID

IIVSLFNGNLVLPKRQETFDLFVKGFNKWVTNGRILLEPVVVNNRPILPTLNDAWFAGFTDGEGCFTCSIGEKRGFSF

NFNISQKWEINLTVLEHFSVLFKSGIVSRYSEENTYEFRLGGVNNCNNVFSYFDKYTLYTKKSLSYKLWKNIHNDLVN

KNHLDESKRREMIERTKMINKSKNTN"

14104 14715 gene

gene orf203

14104 14715 CDS

product hypothetical protein

note LAGLIDADG

translation: "MDTRDERALQAVKNVYGGSIKLRSGVSALRYRLHNKAGLLNLINDVNGH

IRNPIRLIQLNNICVKYDITLNSSKKLILNNGWLSGFFDADGTITINTSNWQLSISAGQKTPEILAPLVELFGGYVYI

DRGGNGSFKWYVTKKEDILNLIEYFKKYPSRSAKNNRLHLVPKFYELKGMKAHIAPSGSLLSKSLDTFINKWNNYE"

>15457 15870 gene

gene orf137

>15457 15870 CDS

product hypothetical protein, partial

transl_except (pos:15457..15459, aa : V)

translation: "VEINRGFSRMVLIDWQDPYGESIRHIYGSFKGFVRVELILPNGEEWTGY

CWPKESEYPSLFKYIPGAIGSGEEAKVYFPAPNNEDWRLANQKYSISSGDNQTVKIADRADVRCVWAEARAEDKACTW

IRPKYVSWKN"

16054 17067 gene

gene orf337

16054 17067 CDS

product hypothetical protein

note LAGLIDADG

translation: "MHPEVTYVGLLTLLYTGTPSLYSSKYSLLNDIVKKLEQRRKSVGNIFYI

KIGTSETIRDGVVVNLENVKRVSDHVPKHFKPLNNKQLGYYLAGLIDGDGHFNKAQQLVIVFSSPDAFLAYYLKEKLG

YGNVRKVKNKNAHLLIVSNKEGMLNVINLINGKLRTEHRFNQVVNNVLSHTKYVDQNINFTVDSSKNLDNHWLAGFSD

ADASFQIKIVKRITRNKPEIRLNFQIDQKSDLLLNMIKEYLGGNIGYRKSQDTYYYGSTNFGSAKRVIEYFDQYHLQS

RKHISYLRWRKVYRLIQDKEHLTDKGLSKILTIKSLINRHEENTTIQDKVLTKI"

>17227 18537 gene

gene orf436

>17227 18537 CDS

product hypothetical protein, partial

note LAGLIDADG

transl_except (pos:17227..17229, aa : K)

translation: "KIRPTLNNIYENILHMLGISNALNTCQSKNFKDIAMSNQQGFEIMSSAI

NFIVFLNLNVFIMKCRGLLEILRDYKRMIVHLEWLRDSPKFCIRINFGSCHASQGQKLINVSLNKLLKRGFSLDIARA

PNNNKPYNTNGIKLDPMWVTGFVDAEGCFSIIIEITDSLKWKVRTSFEINLHEKDAEILYEIQSFFGVGGIYNRSDRK

ISVYRVTNVNNLNDVIIPHFRKYPLISKKGLDFVLWSSVIKIILNKDHLTKAGFLTILSYYASINRGVSKKVLKYYPN

ILPFPKAIINLPDNLNPQWVSGFVAGDGGFSVYIRPAKDYLLLEKVDCRFHIAQHSKDIELMKLFVKFFDCGLVNLRS

NLSTPRCDFIVQDVTSLLNKIIPHFYLYPLLNLKQEDYNCFKEAMLMVKLKKHLTKEGLIKIKSLNLEMNSNRLK"

>18617 19675 gene

gene orf352

>18617 19675 CDS

product hypothetical protein, partial

note LAGLIDADG

transl_except (pos:18617..18619, aa : W)

translation: "WVLASPHSDMWINIIYFAISWNGLVLISTLNCKNLISYTRSAGNLSLYS

VRSNIQSASETIRETSFNFSAFNLYYNTLFGNAPQHLSNNWLTWFIGFVEGDGAIQTYAKGTRVRFVLTQKESAILFY

IQKKLGIGQVKHFPQGKSGDNNDFYRLIVDNPLHILLLAYLFNGNLALTNRIQQLSLWVQALNNRLGADTIILINQAV

SVTLQDAWLSGFTDAEGCFNVSITSNARYTLGSVIKMRYILDQKDSVILMAIQNLFGFGKVSVRSQTDGVYRYTVTGF

KSMNDVISYFKAFPLLTKKAQSFEKWFSIHNMVSNKLHLTEEGLAQVRVLQKQINLENGMTKKTGSAHP"

2166 20793 gene

gene cox1

2166 2268 CDS

4814 4874

6660 6728

7996 8086

10468 10588

12150 12372

15444 15456

17134 17225

18571 18616

19780 20793

product cytochrome c oxidase subunit 1

translation: "MIVRWSRSHLRLKGSGYLVHIRLELSGPGVQYIADNQLYNAIITAHAIL

MIFFMVMPALIGGFGNFLMPLLVGGPDMAFPRLNNISFWLLPPSLILFLFASGIENGAGTGWTLYPPLSGVQSHSGPS

VDLAIFALHLSGISSLLGAINFITTILNMRAPGISLHKLALFGWAVVVTAVLLLLSLPVLAGAITMVLTDRNFNTSFF

EAAGGGDPILYQHLFSRLGIVSCYILIIPGFGIISTVISASSNKSVFGYLGMVYAMMSIGVLGFVVWSHHMYTVGLDV

DTRAYFTAATLIIAVPTGIKIFSWLATCYGGSLQLTCSMLFALGFVFMFTLGGLSGVVLANASLDVAFHDTYYVVAHF

HYVLSMGAVFALYSAWYYWIPKILGLDYNPVLGKVHFWVLFIGVNVTFFPQHFLGLQGMPRRISDYPDAFAGWNLISS

FGSIISVVATGLFLYIVYVQLVEGKATTRYPWLTPQFYSDSLQTLLNRSSNSLEWSLTSPPKPHAFVSLPLQSSFTEA

FSSIPEYYSWCVDADKHLDTLRTAIHLGSYFNRTMTAFRPDIKRSLTFTHEWAGHLSDDFDPEVVSSSTVIHALSLLN

RHKQELSQLTESDLT"

2166 2268 exon

number 1

4814 4874 exon

number 2

6660 6728 exon

number 3

7996 8086 exon

number 4

10468 10588 exon

number 5

12150 12372 exon

number 6

15444 15456 exon

number 7

17134 17225 exon

number 8

18571 18616 exon

number 9

19780 20793 exon

number 10

2269 4813 intron

number 1

note Group II

4875 6659 intron

number 2

note Group IB

6729 7995 intron

number 3

note Group IB(3')

8087 10467 intron

number 4

note Group IB

10589 12149 intron

number 5

note Group IB

12373 15443 intron

number 6

note Group ID

15457 17133 intron

number 7

note Group IB(5')

17226 18570 intron

number 8

note Group IB

18617 19779 intron

number 9

note Group IB(3')

1. LGKQ01002444.1

>Non-coding DNA

1. LGKQ01002252.1

>Feature LGKQ01002252.1 Table

90 1109 gene

gene orf339

90 1109 CDS

product hypothetical protein

translation: "MQTALIIVNKNSNNNTNMVNYRAFSSVSYSKSVVNNNLVIRSKNQTALI

LRVDIKPNLKLITLDVRHFSSTSSFSNLNKKKLGLIFTKQTLFKHFSKKDVCYGLVVIIIIASIRYSGVVEIILVFLF

DSSPEWAQLALASALVLPLKLGLKGVVGDISDVFDLEKDKLTMGGDKPNIKSGFKQIDSKNLIGNAMNTSDSGGGSSQ

QGSGGDSSQQGQRARVRRIWNVNYIQPHQVEMMIASARYRIDYLERRIAYLSNLRPEGIELIEYAKLKLECSKLKQEL

IQTREDLRGIHSELQMHGLLSSSRYTGEKWLRYYREAAEQSGNVRPMTNRNNVDLD"

4. LGKQ01002291.1 (reversed)

>Feature LGKQ01002291.1 (reversed) Table

11 808 gene

gene orf265

11 808 CDS

product hypothetical protein

translation: "MKRQFKTRKNYANVRLSYLTASKSRLFGANIILRDRKVREFTVSSGFNT

ENPPLSEKAMGKRPATEDNSVSNTEMNTMDNPFGSKNIHILDATATRAAASYEDSYTGNNISEDKEFQKLQYKSWMYS

AAKALHDSLPDDDERKNSRNVDALIKAVTDKTQPNCEAEVFGLSEMVDKTVHDIPKWPTLTSFFGNKRKFEEDGESST

QPSKSIKESFSHITNNSPEDPTAKSSVANPENKNLDNRSPLDFVLDKQQSEPFDFTDSEE"

1. LGKQ01002358.1 (reversed)

>Feature LGKQ01002358.1 (reversed) Table

415 485 gene

gene trnW(tca)

415 485 tRNA

product transfer RNA Tryptophan

anticodon (pos:447..449,aa:w)

1. LGKQ01000814.1 (reversed)

>Feature LGKQ01000814.1 (reversed) Table

127 3290 gene

gene nad4

127 642 CDS

2340 3290

product NADH dehydrogenase subunit 4

translation:” MLLLLLLIIPLLGVFTISTGISYNLSDLNIRRIKKIALTASIINLFLSFLIFILFDASSNQFQFVQEYHEISSFDFYLGLDGLSIYFVLLTTIITPIALLSNWKSIQDNVRSYVIIILLLESLLLAVFLVLDILLFYIFFESILPPLFILIGLFGSNNKVRASFYLFLYTLFGSLFLLLSILTISSIMGTTDFDALYKTNFYYSTQLFLFYGIFIAFAVKTPTIFLNTWLLKAHVESPLGGSIILAAIVLKLSLYGIFRLMLPLLPKASLDYTYIIYLIGVITIIYASFSTLRTIDVKELIAYSSVSHAAVYLIGVFSNTIQGIEGGIVLGLAHGFVSSGLFICAGGVLYDRSATRLISFYRGIAQLMPLFSILFFILSLANAGTPLTLNFVGEFMCLYGTFERLPLLGLFASSSIVFSAAYTIYMFNRIAFGGSYSKFFEVNISDVNKREFFILLTLVVFTVILGIYPAPILDGLHYSVSSLIYSNLS”

127 642 exon

number 1

2340 3290 exon

number 2

643 2339 intron

number 1

>948 1433 gene

gene orf161

>948 1433 CDS

product hypothetical protein, partial

note LAGLIDADG

translation: "-IVMWLMGRHDRAFIHTTRSIQNLESLTSLLNKECEQVNNISTLVKPTA

LNITKLDPWFITGFVYAEGCFMLGMFVSSKYRMAYQVQAIFKISLDHKDYDLLCKIQDYFGVGSITKHGSTSLQYTIK

SLKDFSIIISHFDKHPLISQKKGRITNFENQLLH"

1496 1963 gene

gene orf155

1496 1963 CDS

product hypothetical protein

translation: "MNLGLSDELKLVFPDIRAVSRPLLKDKDVKDSNWLAGFTNGEGCFYISI

LKSSTCKIGKAVILKFQIAQHSRDTELMKSLISTLGCGRIELNLARSTVNFLVTKYTDISDKVIPFFDKYPLFFEEEG

AKVSDYNYFKQVSALKSKKAHLTEQGLS"

5064 5618 gene

gene orf184

5064 5618 CDS

product hypothetical protein

translation: "MGFRNFTTYLSKNSAAFGSVSDNFTNKDFLFWFSGFTDPEGNFLVTIDR

KYVKLRFKISLHIDDIEVLYTIKSNLGFGRVVEEYNRNSCSFIVEDFLSISKLCDIFNHYPLHTSKKLDFLSFYEVLL

IKSTKGLSEVDIKKIVSIKNTMNSKREVFTYGISESQIIINPNWFIGFIEGEGTLLR"

6028 6753 gene

gene orf241

6028 6753 CDS

product hypothetical protein

note GIY

translation: "MIMRDVDCTGFFDNNLVYLFLVGGICYYTAYSFAKLYENADTQKSFILG

ENRDRSGIYLWRNLVNGNMYIGSSVRLRIRLLQYYNAEYLERNSSMIICRALLKYGYSNFSLTILEYCEPEECLVREK

HYIDLMKPEYNFSQNPSSPWLGLKHTIEARAKRSTNTLGLSKSEEHKLKLSLADPRSVVVLVTDLTTNISTEYNSMSA

AAKALGIGKASVIHYFSNNQKKPYKGRYLFSKVEKE"

6782 7228 gene

gene orf148

6782 7228 CDS

product hypothetical protein

translation: "MALLLKIYGYYYTIEGKNLFLDISEILNKRYSTNSSVSDINNVIKNITE

KFNDIMQKDSPFDVKLNLRHTENVRKYSIANKSENSKIVYIYDGNEMVHGSPFASFSAAHKALGLNPSSNTCNRYIDT

NRLYKSKYIFTSKPIDRASRD"

>7468 8490 gene

gene orf340

>7468 8490 CDS

product hypothetical protein, partial

note GIY

transl_except (pos:7468..7470, aa : Y)

translation: "YFYSQRWFNIRIAVIFIIIVYNMIQNLCVNSRTHLIRNIYNLKYNKLNN

KSLQGKLRFRLNHFSKKMYSTSPCLSKSSCSETGSEKLGTIIKELGLNPVYIFENLNSENVRKQILDKTRGLSGIYII

VNKITKDYYIGSASTSRFYARFSNHLIFFRGSKIVKLAVKKYDLNNFAFIVLELYPHIVTKENNKELLDLEDKYLKLL

LPNYNILTEAGSSFGYKHTEVARKKMIDLYSEERRERIGSLNKGKKLSPETIERIREKALNKRPMPDEIKKKCISNTR

PIILYNLNDTVYGEYSTILEAAESINCNEKTIRRALKTPKKIVKRQWIVQDFSTTFK"

>8874 9638 gene

gene orf254

>8874 9638 CDS

product hypothetical protein, partial

transl_except (pos:8874..8876, aa : I)

translation : "IISCLTCNALWYECLVLFLFAFFLSSILLINVYLGPNSWRQGKLANRIP

ANIRIGPHNHDILSILTGTLLGDAHAERRRSGNGTRISFSQESSHGEYLHYLHGLIAKLGYCNPAVPVIQTRLVAGGK

IRYTLRFHTYTYTSLNFIRDLWYDSKGTKVVPNTIADFLTPLALAIWIMDDGGRVGYGLKLATNSFTFADTTRLVQIL

HNLYGIKATVQSAGVSNQYVIYVWSESMPWLRELVRPYMVSSMLYKLGE"

>10109 11008 gene

gene orf299

>10109 11008 CDS

product hypothetical protein, partial

note LAGLIDADG

transl_except (pos:10109..10111, aa : I)

translation : "IFDQFKHFTFFSDLSTIAQFGTGLPVIGTINYKSRTSKLNRLSTTEYLA

IPKSFLAFLIGFIDGDGYIGIQKNYSGSITVHLTISLHIDDITILNYIQSVLKLGKVYAYPERKTPSVRLIFTKSELQ

EVLFPLFFHHGLFFLTNTRRAQYYMAIHVFNNNVNKYLDLPSVAPVIQELPTSALEYTHLGFFKDWVVGFVCAEGSFL

VKQNNDGCFQIKQRLHLLLFESFKLVFNTNRKITVDKELYLQFGVSSKADIQNVIKFFSFSGYHPLIGLKNIQYSSWL

EKLRNSERYKNLKFPV"

>11570 12529 gene

gene orf319

>11570 12529 CDS

product hypothetical protein, partial

note LAGLIDADG

transl_except (pos:11570..11572, aa : Y)

translation : "YTDEPHCGNVVLKILLNAGTSPNLGFAYYLLLIFISIIYVEIAMTWRQS

AGVRSIHTSEASQRLHAEDLTYAYLVGLFEGDGYFSITKKGKYLTYELGIELSIKDVQLIYKIKALLGVGVVSFRRRG

EIEMVTLRIRDKNHLKNFIIPIFEKYPMFSNKQYDYLRFKSSLLSDIIYSKDLSEYIRSSESLNSIESILDKSYFPAW

LVGFIEAEGCFSVYKLKKDGDYLVASFDIAQKDGDILISAIRKYLSFTTAIYLDKTNCSKLKVGGVRSVENVVKFLHK

APVKLKGNKKLQYLLWIKQLRTIPRYYEKFKIPPVY"

13458 14738 gene

gene orf426

13458 14738 CDS

product hypothetical protein

note LAGLIDADG

translation : "MTLPEVKSIFLTLKNSGPSGKGSDKEFCLLANGFWQAEGYIGGIFRSEL

NFYPLCTATQLFSVESAEFFIRLDKALSNKGTFSITLNSFGKFVIAYRLSGWDTFFSVFVPYFHMLYGEKYQAILKLK

EIHALKPLIKDNTDNMSKVLLVSLVYSLTAHSSRYKVSLEDKLLSLGLDLALLKELPKVSYKENVIKPSFLFILGFFL

GDGTLQLKLEWKERNSTVVVVPLFNIVQSNIESNKYIMETMANALNALNIKTSLEKSVNTYTLTVKGIHNVFNSLFPL

LKNYSHFLYGKSESFNLLVWVERLVRSGGHHTYFGLRALINKMYDSTNERYTDKEVWMGRIEDWLKAVSARREWGEYF

ISPIYTPNREIRGWQVRFPSTLKFLKSNKAFILRGKEEPAKALLSAVEYRDKILSSWIDKLFRGG"

14845 15174 gene

gene orf109

14845 15174 CDS

product hypothetical protein

note LAGLIDADG

translation : "MLIPFTSLLEGTDSVRSKFSGSTLHPLWVTGFSDAEASFALDFAKKAGS

PAGWQVLPVFKINLHAKDLALLQQIQVYFNNLAQESGQTCSGRFWVSRDIASLASLGLVL"

15273 15578 gene

gene orf101

15273 15578 CDS

product hypothetical protein

note LAGLIDADG

translation : "MSTKQHLGHGGLDKIVNIKASLNLGLSEELQAVFPNNIKVNRPVVDNKS

IPHGMWMAGFTSGEGCFLVSIFKSTTTKLGYTPRLRFSITQHSPSERWTTFT"

4605 16476 gene

gene cob

4605 4805 CDS

7276 7467

8838 8873

10040 10106

11559 11568

12631 12903

16080 16476

product apocytochrome b

translation : "MRIFKSHPLLRLVNSYMIDSPQPSNLSYLWNFGSLLAVCLVIQIITGVT

LAMHYNPSVLEAFNSVEHIMRDVNNGWLIRYLHSNTASAFFFIVYLHIGRGLYYGSYRAPRTLVWTIGVVIFILMIVT

AFLGYVLPYGQMSLWGATVITNLMSAVPWIGQDIVEFLWGGFSVNNATLNRFFALHFVLPFVLAALALMHLIALHDSA

GSGNPLGISGNYDRLAFAPYFLFKDLITIFLFIIILSIFVFFMPNVLGDSDNYIMANPMQTPPAIVPEWYLLPFYAIL

RSIPNKLLGVIAMLSAILILLAMPFTDLSRSRGIQFRPLSKIAFYIFIANFLILMVLGAKHVESPYIEFGQISTVIYF

SHFLIIVPFISLLENSLVELAVLTKEKPSR"

4605 4805 exon

number 1

7276 7467 exon

number 2

8838 8873 exon

number 3

10040 10106 exon

number 4

11559 11568 exon

number 5

12631 12903 exon

number 6

16080 16476 exon

number 7

4806 7275 intron

number 1

note Group IB

7468 8837 intron

number 2

8874 10039 intron

number 3

note Group ID

10107 11558 intron

number 4

note Group I(derived)

11569 12630 intron

number 5

note Group IB

12904 16079 intron

number 6

note Group I(derived)

1. LGKQ01002032.1 (reversed)

>Feature LGKQ01002032.1 (reversed) Table

1604 2146 gene

gene orf180

1604 2146 CDS

product hypothetical protein

note LAGLIDADG

translation: "MQLKEHNTLKGLQKIINIRATLNFGLSRELQLMFPETIPVPRPLRETCV

MPHSDWIAGFTSGEGNFSVSLDKGIFKSLLFKITQHERDEVLLTAIKEYFNCGYCYLRKQENTIDFKVTKFTDLNKII

IPFFINSPILGVKSLDFKDWCLVSEMVKKEEHKLKEGAIKIREIQRGMNRGRS"

2868 3392 gene

gene orf174

2868 3392 CDS

product hypothetical protein

note GIY

translation: "MPTEVCKPIIEFNSLDDNLVVLSYREVLKNKGGVYCFINTVNGKLYIGS

AKDLYLRLSEHLSNRKSNVVLQSAILKYGLDKFNFSVLKYFTYDSKEVSHKALTDLETSYIEKYSFDCLYNFKQTATS

STGYKHTDEAKLKMLRWYENKSNHPMFGKTHSAFRRVHVCLSVNLDN"

591 4103 gene

gene nad1

591 881 CDS

2177 2521

3648 4103

product NADH dehydrogenase subunit 1

translation: "MLYLPTLISVIEVLLVTVPVLLTVAYVTVAERKTMASMQRRLGPNAVGY

LGLLQAFADALKLLLKEYVSPTQANLALFFLGPIITLIFSLLGYLVVPYGPGLALSDINLGVLYMLAVSSLSTYGILL

AGWSANSKYAFLGSLRSTAQLISYELILSSAILLVIMLTGSLNLTVNIEAQRAIWFIVPLLPIFIIFFIGSIAETNRA

PFDLAEAESELVSGFMTEHAAVIFVFFFLAEYAAIVLICIVASMLFLGGYLFNIMPLVYLVQFFDFDFWLDLTNNGRD

TYVILDSVIEGLLYGLSLGVKSCALIFTFIWTRASFPRIRFDQLMSFCWTVLLPIVIAFVILVPCILYSFDIIPSNIT

LF"

591 881 exon

number 1

2177 2521 exon

number 2

3648 4103 exon

number 3

882 2176 intron

number 1

note Group IC2

2522 3647 intron

number 2

note Group IB

4381 4527 gene

gene atp8

4381 4527 CDS

product ATP synthase F0 subunit 8

translation: "MPQLVPFYFINQVTFAFILLIVMIYVFSKYILPRFVRLFISRVFISKL"

4780 5121 gene

gene orf113

4780 5121 CDS

product hypothetical protein

translation: "MPRFVMLIFIHYLVVCGLLARENNMIFLKQCDLIFFAPHTSVGFNNNRG

GSYNNRASTRNDSKNTASYTNTKNILIIIITLLIVLVLAFVLLKILLDEAILCDKAIIFNKLYT"

5173 7611 gene

gene atp6

5173 5744 CDS

7404 7611

product ATP synthase F0 subunit a

translation: "MFNLNYTQIPSPLDQFEIRNLLSLDAPILGNLHFSLTNIGLYLTIGGLL

ILTLSVLTTNYNKIVSNNWSISQEAIYTTIHSIVTNQINARSGQIYFPFIYTLFIFILINNLIGLVPYSFASTSHFAL

TFALSFTIVLGATILGFQKHGLEFFSLLVPAGCPLALLPLLVLIEFISYLARNISLGLRLGANILSGHMLLNILSGFT

YNIMTSGIIFFFLGLIPLAFIIAFSGLELGIAFIQAQVFVVLSSSYIKDALDLH"

5173 5744 exon

number 1

7404 7611 exon

number 2

5745 7403 intron

number 1

note Group IC2

1. LGKQ01002076.1

>Feature LGKQ01002076.1 Table

471 1088 gene

gene atp8_t

471 1088 CDS

product truncated ATP synthase F0 subunit 8

translation: "MPQLVPFYFINQVTFSFILFIVMIIFVLYMTPQAQENHIVFVALGFFFL

FVCFSVLIAIFHTPAQLDTMNVSWGPNFDDAKFFVLDTCSIDKPDLVVDTLYNGSSLGELKTLIENFFSTVRFKKIAL

DIFSKSIPGKKVPLLGIKPIGLIKLKISPSFVKCSSGISRGHVWPILRKNPLPLLLKSHFVFFFXXFFFLYSFITIYL"

2030 2099 gene

gene trn*(uua)(tta)

2030 2099

product

anticodon (pos:2062..2064,aa:*)

3923 3994 gene

gene trnK(ttt)

3923 3994 tRNA

product transfer RNA Lysine

anticodon (pos:3955..3957,aa:k)

1. LGKQ01001821.1 (reversed)

>Feature LGKQ01001821.1 (reversed) Table

67 3233 gene

gene nad2

67 447 CDS

1920 3233

product NADH dehydrogenase subunit 2

translation: "MVIFSILFLLLSNAVTLRRDKSILFSRVAITVLLYSSLIAFYSLYFCLDSGIGLYGGLFHATTTTHVFHIFIFLISAAILQLTAFYPRKVWIAEYSSISKLLLYNFLYYRSKIVNKMGEQFKIIEYPLILLFIITGAIFLVSTSDLVSIFLSIELQSYGLYLLSTLYRNSELATSGGLTYFLLGGLSSCFILLGSSLLYANSGTTILDGIYVITSLSDIGNNGQASAENLLYWYKPYYINFSLLIMSVGFLFKVSAAPFHFWSPDVYDAIPTIVTTFVAIVAKISIFIFFLELVHYTSNSLFSFQYNWTTSLLVSSLLSLVIGTVVGLTQLRIKRLFAYSTISHVGFILLALSINSIESIQAFIFYLMQYSISNLNAFIILISIGFSLYYYVNDNEEYKELVDKNNSPIQLISQLQGYFYINPVLALSFTITIFSFVGIPPLIGFFAKQMVLSAALDSGYVFLTLVGILTSVISAVYYLNIIKQVFFEKPEYKINPEIVNINLHGSIIKKNVLIKKLTFKLDNIVLSSSLTITISVLTLIILLFIFTPQEWLSMANILALILFNV"

67 447 exon

number 1

1920 3233 exon

number 2

448 1919 intron

number 1

950 561 gene

gene orf129

950 561 CDS

product hypothetical protein

translation: "MNQGFKLRLVSPIVDNLAGIRFFELLVVEYSRLFRIWLDGIPAQVILSN

PLRVYLKFTGHDPVNQLPSTRCSFTNMLFDLDPRLSIDLSTEKIVFFLPLRNNPRYIPERNFLVNLRIQRVIFWVISV

PE"

3237 3698 gene

gene nad3

3237 3698 CDS

product NADH dehydrogenase subunit 3

translation: "MTSTTFFFIFIPILAVILLAVNLIFAPHNPYQEKDSVFECGFHSFLGQN

RTQFSISFFIFALLFLLFDLEILLVYPYVVSAYTNGVYGLVVMLIFFLALTLGFAFELGKNALKIDSRQNLQFLNLEI

DRIYSYCHEHTKHSAVQPARAKKVYK"

1. LGKQ01002440.1 (reversed)

>Feature LGKQ01002440.1 (reversed) Table

809 444 gene

gene orf121

809 444 CDS

product hypothetical protein

translation: "MNVKISRLTQARDYHKASTLFMKMLSHSKSLRVYAIYQFFRGWYYGKPY

YQVIRVANRFHKFMITLPQFVTMKRRYIRRDSERGDHYEYLTWQCVYWTPCERNIYICQHKALAGTHSTRIP"

1. LGKQ01002223.1 (reversed)

>Feature LGKQ01002223.1 (reversed) Table

26 1282 gene

gene orf418

26 1282 CDS

product hypothetical protein

translation: "MLLLLLLIIPLLGIFTISAVISSNLSDLKIKKFLKKKLVFYKNKVFLFL

VTKSKINETYIIPSKGNLYIIFIWLSSVLILPFFLLNIIILIDIDTDINLNNLIILIFFSLSQTYICTLAGLNFLLGN

NYNTWKVLFNIWVGLEKNKWSYLFQIFLFILLVVICASVLINSSIYFLDLNSCNFITYLWLRLSLAPYLIYILNIITT

IYLKIFRLNKPELDLSIFSPNMFKAITFNRLLVMSISISLFFYFKLIVISKIILDFSFDIDFWVGILLAIFNRIIMLS

IDLFDAAKPWSVAHAEGPDSLEGEEPDSLEGEEPASLEGEDTNSLEEVKRLRRERRAKQMRERRARLRELYNETPARK

EKLAQKRAVREEKERIKWSKAKSKEIEAWYRNTTTLSPIILPASLAPERVNYIRTIPT"

1. LGKQ01001994.1

>Feature LGKQ01001994.1 Table

251 430 gene

gene nad3­_t

251 430 CDS

product truncated NADH dehydrogenase subunit 3

translation: "MAQNRTQFSISFFIFVLLFLLFDLEILLVYPYVVSVYTNGIYGIVIMLI

FFFSINIGFCL "

1653 1952 gene

gene orf99

1653 1952 CDS

product hypothetical protein

translation: "MAILDNTLGGDGKIHSIFESDLTEVINGLEEGSKKKNFFFEKRPFSVEL

FYNLDSKNKNRFNLDKFRSGYSNLLLTVFIMFYIRIIFQIIYIIRHLKLF"

2238 2993 gene

gene cox2

2238 2993 CDS

product cytochrome c oxidase subunit 2

translation: "MLNLLNLNLLNNCDTPRPWGLYFQDSATPQMEGLVELHDNIMFYLVIIL

FGVGWIMISIVRNYTSVNSPISHKYLNHGTLIELIWTITPAIILILIAFPSFKLLYLMDEVSDPAMSVLAEGHQWYWS

YQYPDFLNTDNEFIEFDSYLVPESDLEEGGLRMLEVDNRVILPELTHVRFIVTAADVIHSFACPALGIKCDAYPGRLN

QVSVLINREGTFYGQCSEICGILHSSMPIVIESVSIEKFLSWLEEQ"

4388 4458 gene

gene trnW(tca)

4388 4458 tRNA

product transfer RNA Tryptophan

anticodon (pos:4420..4422,aa:w)

1. LGKQ01001551.1

>Feature LGKQ01001551.1 Table

1355 1622 gene

gene nad4L

1355 1585 CDS

1587 1622

product NADH dehydrogenase subunit 4L

translation: " MSLSLILFLIGILGFVLNRKNIILMLISIEIMLLAITFLILISSLSFDDILGQTYAIYIIAIAGAESAIGLGILVAFYRLRGSIAIEYK”

>1874 3418 gene

gene orf514

>1874 3418 CDS

product hypothetical protein, partial

note LAGLIDADG

transl_except (pos:1874..1876, aa : Y)

translation : "YFVVIFFYLWEIEAVLVWIQLYKLIFIQFLKGLFFFISSWIISPHRAKD

RGADLLYSLQSIRGAATTYKTISSSCIGEAIALRRNYTSASFLLRDVNLNVTTLSTQDSNFLQWFVGFTDAEGNFIIN

RILKKDKITTSSFSFMFKITLHKDDEMVLRYINNKLGVGGVRFYKDECIFNVTDKKGVALLIDIFDKYNLNTTKHLDY

LDFKEAFNFYSNRSKNLKPDVVKDYLLELKNKMNTNRIHFERPIKSEINITKSWLLGFIEGDGSFFLRRDNIIPTFSI

ELTGVQFPIMLKIKEFLENSLGFDPYSLYKLKNSSIIAITTVKARNNAKSSVSLIIKNINVLNNYFIPFFADSEFLTQ

KGLDFHDFKIISQAVFIGAHRNEEIRSLILNLSNTMNNFRLSTYKGTVQVLSIEKIKQITMAVPTIEYLLDGRVIDRS

TKKLLARLVTCVYEIYNADESIVLANSLTEAASIVGLYPDTLSKYLDVEVLNSEGTFVNIKNNKIRRVRVFLSIK"

>3999 4469 gene

gene orf156

>3999 4469 CDS

product hypothetical protein, partial

transl_except (pos:3999..4001, aa : R)

translation : "RGCVRGKRIYGDKLSNSGNLLKLKVPSYNWKVISGRINYSGMVISLKMS

ENEMDYRGSKLIILNSVSVKEQRVDGSWSIKPHLINLRCTLGGFERNRGVKLGFNMQQGWNSYVKIPSKQFDLKKFST

CDSTHACAVNPGGLIWFNRWWGFIWYNSR"

4795 5160 gene

gene orf121

4795 5160 CDS

product hypothetical protein

note LAGLIDADG

translation : "MNLGLSEMFKSEFAGYTPVERPVINSDNVYLDPDWISGFVSAEGNFDVR

MPSTNSKLGYRVQLRFRVSQHSRDLRLMEKIVEYFGSGKIYKYGGKSTVSLTILDFTDITNVIVPFFNKTLL"

>5607 6575 gene

gene orf322

>5607 6575 CDS

product hypothetical protein, partial

note LAGLIDADG

transl_except (pos:5607..5609, aa : R) translation : "RNSKLLKVYLNRLNKNQIQKILPYRQVRINSGYTVSANFFMASYLAGLI

EGDGHIAVHDKNSSSKKYRPKIIITFNLADKSLADKLSAILKVGKVISKPSAGHVILQILAKDEVLKIINLINGYMRT

PKIEALHRAITWINEKDSSSIPCLGLDLSAIDSNSWLAGFTDADGNFSITVTDVKKKGIFKNKRVQTFFRIEVKQNYS

REVTEAQGGGYFYILTKITAFFTVNLYTRTRYVEDKVYYSFMAIAHNSRSHEIVRKYFDAFPLYSSKYLAYKDWCYVQ

DLLKVPLNKEGLDKINEIKAQFNSKRKVFDFSHLDSLTF"

>7419 8333 gene

gene orf304

>7419 8333 CDS

product hypothetical protein, partial

note LAGLIDADG

transl_except (pos:7419..7421, aa : L)

translation : "LYRAFLKLHYMREHFVSIFWSTVYFVSFGKIQNEEQSAGNQINNLGSSE

TTCEAITLSDKEKFKWWLIGFAEGDGNFSVDKIGYLTFKVTQSSVDAQVLFYIKKELGFGSVTEQSNLNQTHQFRVRS

KENLLKIIDIFNGNLITKAKKAQFKSWLEAFNNKYGTNIIHIESDKKVTLCNAWLSGFTDAEGCFTSSAFLNKNTGKH

IVTVRYVISQKDDLEFSTYLAGLIDGYVTYVKSYDGYNTVVNFSKLNKALSYLHNYSLKSKKHLSYLKWLKVYNLVKD

KKHLTDSGIKIIKEKIKLINK"

1625 9703 gene

gene nad5

1625 1872 CDS

3923 3998

5361 5606

7272 7418

8435 9703

product NADH dehydrogenase subunit 5

translation: "MYLAIITLPLLGSIVAGFLGRKVGVSGAQFITCSSVIVTTLLAIVAFFE

VGLNNIPVSINLFRWIDSESLNVLWGFHFDSLTVSMLIPVLIVSSLVHIYSIGYMSHDPHNQRFFSYLSLFTFMMIVL

VTANNFLLMFVGWEGVGICSYLLVSFWFTRIAANQSSISALLTNRVGDCFLTVGMFAILWSFGNIDYATVFSLAPFVS

ENIVTIIGICLLIGAMAKSSQVGLHVWLPMAMEGPTPVSALIHAATMVTAGVYLLMRTSPLIEYSSTVLILCLWLGAI

TTVFSSLIGLFQQDIKKVIAYSTMSQLGMMVIAVGLSSYNVALFHLVNHAFYKALLFLGAGAVIHSVADNQDFRKYGG

LRPFLPLTYSVMLIASLSLVAFPFMTGFYSKDFILESAYGQFYFSGTVVYFIATIGAMFTTLYSVKVLYITFLTNPNG

PLINYKNAHEGDLFLSIPLIILALFSIFFGYITKDIFIGLGSGFFADNSLFIHPTHEIMLETEFAVPTLFKLLPLVFT

LSLSTLAIILSEFIPTALVHFKFTRLGYNLFGFFNQRFFIEMFYNKYVTNFVLNTGGITTKFLDKGSVEMIGPYGLEK

GLLKLSNNIESLSTGIVTNYALYVLIGFVSYLLIIFLELNMDLVLLVLLIIPFSLMPISMQNIHRY"

1625 1872 exon

number 1

3923 3998 exon

number 2

5361 5606 exon

number 3

7272 7418 exon

number 4

8435 9703 exon

number 5

1873 3922 intron

number 1

note Group ID

3999 5360 intron

number 2

note Group IC2

5607 7271 intron

number 3

note Group IB(3')

7419 8434 intron

number 4

note Group IB

1. LGKQ01002591.1 (reversed)

>Non-coding DNA

1. LGKQ01001556.1

>Feature LGKQ01001556.1 Table

2343 2588 gene

gene rnpB

2343 2588 ncRNA

product ribonuclease P RNA

2728 4276 gene

gene rns

2728 4276 rRNA

product small subunit ribosomal RNA

4621 4705 gene

gene trnY(gta)

4621 4705 tRNA

product transfer RNA Tyrosine

anticodon (pos:4656..4658,aa:y)

5225 5295 gene

gene trnN(gtt)

5225 5295 tRNA

product transfer RNA Asparagine

anticodon (pos:5257..5259,aa:n)

5318 5389 gene

gene trnR(tcg)

5318 5389 tRNA

product transfer RNA Arginine

anticodon (pos:5350..5352,aa:r)

5646 6320 gene

gene nad6

5646 6320 CDS

product NADH dehydrogenase subunit 6

translation:

"MINLILLWEIFTNSFRAEMLDVASLFAIFCAILVIVSKNPIVSALFLIG

LFLSISCYLIMLGINFIGLSYLLVYVGAVSILFLFILMLINVRISELLTDTSNSLPLAIIISISFYSSVHSTIPYSIV

PFTSYISNISNTFNDILDMILLYINNGIISFTSNFTNTSFTTTNIWDGNLSETSHITSIGNIMYTSYSIWLILTSIIL

LLAMVGCIIVVIKVDKKEE"

6948 7019 gene

gene trnV(tac)

6948 7019 tRNA

product transfer RNA Valine

protein_id lcl| G-trnV

anticodon (pos:6980..6982,aa:v)

9453 10121 gene

gene orf222

9453 10121 CDS

product hypothetical protein

note LAGLIDADG

translation: "MYAFIQSELGNVGRFQITGENILRYIIGDKAGIMLFINLIHGKLRTPKN

KRFNDLIKVFNVKYSLGISESLLDNSDFTNNSWFTGFTEADGHFGIKYVESKAKSDTRKRSVSENISLKFRLDQRSYD

KPTSSSMKPFMESLALFLSCNLKNYTNNKGSEALSLSVLSINGVKFLIDYFNKYPLLGNKSNDLNKWEIVYNMIISKE

HLTEQGRLKIKSLIGKL"

>12109 13554 gene

gene orf481

>12109 13554 CDS

product hypothetical protein, partial

note LAGLIDADG

transl_except (pos:12109..12111, aa : K)

translation: "KINTTDILYISIAVWVEISLYTFIIHIMFSSNYFYTIFIKSLKPKFHVA

CLSISRSISFGSGSRASQMQFSTSNLNVEFYKWFSGFTDAEGTFMIVSSIKGFSFKFSIGLHIDDLNVLNNIKDKLGF

GNIYISNNTCHFNVTKKDDILKLINIFDIYLLNSTKRFDYLDFKKAYYLYKNRDELTQELTNQILDIKSNMNNSRKFL

ESLLLSPNPEDRDTWNAEFKISKEWLLGFIEGDGSFSLSRNTMEPVFSIKLSESQRSLLNAIKEYLKNNLGLDTYSLN

KLECSSVISIGKGKAVNNSKPLATLTIKNTQFLNNVFIPFFDEMKFISKKGLDFKDFKLICHAIFIGAYRTERIKGLL

IKLSMTMNNFRLSDYKGEKVNISLAEILEILDAEATIEHFSDGRELDINTKKLIHRRSSSSVFEILNPSGEIIIKSNL

ADSAKEIGVGFNTLKRQLDNRIQEVEYKQYKIKRIGVFKNKM"

14046 14615 gene

gene orf189

14046 14615 CDS

product hypothetical protein

translation: "MERDGNGSRFCFYQKGEHIEYIIWLHSVLLKHGYCKENIPQIQSRVING

KLNYYCRFRTFTYSSFNWIYDDFYPLSPSGQEGQKVVPDWIEEYLSPMALAIWIMDDGGWIKNRGVKLSTNCFTLKEV

KLLVSILEKKYSLSIAIHSAGAIDQYNIYFPKKNLPILIPLVLLHMHPYFLYKLNMVKPNIF"

7646 16933 gene

gene cox3

7646 7754 CDS

9075 9184

10364 10477

12013 12108

15020 15230

16764 16933

product cytochrome c oxidase subunit 3

translation: "MTILTRSNFQAHPFHLVSPSPWPLFTCIALLTLTTTVVLTIHGFSNADY

FLTIALISVVSSMSFWWRDVISEGTYLGNHTLSVQRGLNMGVALFIVSEALFFLAIFWAFFHSALSPTIELGAQWPPM

GIEAVNPFELPLLNTVLLFLDLVTVTFAHHSLIQGNRSGALDGLVATVLLALVFTGLQGVEYTVSSFTISDGAFGSCF

YFGTGFHGLHVMIGTAFIAVGLWRVLAYHSTDNHHLGLEASILYWHFVDVVWLFLYISIYYWGS"

7646 7754 exon

number 1

9075 9184 exon

number 2

10364 10477 exon

number 3

12013 12108 exon

number 4

15020 15230 exon

number 5

16764 16933 exon

number 6

7755 9074 intron

number 1

note Group IB

9185 10363 intron

number 2

note Group IB

10478 12012 intron

number 3

note Group IC2

12109 15019 intron

number 4

note Group ID

15231 16763 intron

number 5

note Group IA(5')

19269 19340 gene

gene trnK(ttt)

19269 19340 tRNA

product transfer RNA Lysine

anticodon (pos:19301..19303,aa:k)

1. LGKQ01002074.1 (reversed)

>Feature LGKQ01002074.1 (reversed) Table

93 172 gene

gene trnS(gct)

93 172 tRNA

product transfer RNA Serine

anticodon (pos:125..127,aa:s)

237 307 gene

gene trnW(tca)

237 307 tRNA

product transfer RNA Tryptophan

anticodon (pos:269..271,aa:w)

672 876 gene

gene cob_t

672 876 CDS

product truncated apocytochrome b

translation: "MIIFKSHPLLRLVNSYMIDSSQPSNLSYIYNFGSLLAVCLVIQIIVGIT

LVMYYKPSTLGIFNLVEHI”

1867 1938 gene

gene trnI(gat)

1867 1938 tRNA

product transfer RNA Isoleucine

anticodon (pos:1900..1902,aa:i)

2051 2136 gene

gene trnS(tga)

2051 2136 tRNA

product transfer RNA Serine

anticodon (pos:2085..2087,aa:s)

2178 2250 gene

gene trnP(tgg)

2178 2250 tRNA

product transfer RNA Proline

anticodon (pos:2211..2213,aa:p)

2282 8856 gene

gene rnl

2282 8856 rRNA

product large subunit ribosomal RNA

3155 3469 gene

gene orf104

3155 3469 CDS

product hypothetical protein

6393 8138 gene

gene rps3

6393 8138 CDS

product ribosomal protein S3

translation: "MGKLKQKISQIKPTILFTPHKQARKTNGSKLKDLFNPYPNSVDMGQKQD

AKPLIFKKKQKDVKVIPLKVKTSDTGRIRHFTPAAQEWRNSIYAYNKNYLKLLPVADNNFMSLVKSYFNFYFKNKKSK

KKAKKSNKKGQQNMSRRKRSRSRGLSANKIFVAKGDLKHTSSKAVITLYFYNTEKKFLIRKIKKQIFDLYRPNKRLKR

FINMDRNSKEIITYNRPFSLKEYLRIPNHYTDYVTLFKKSFVEKLCKYFDLTNKHIYLISNLVENKVLNEDEKLLIFK

RKLNSLIRIKYPDYTDYMRKVKLHYLNKLTICIKLLTLNQIKFKHAFLLKLRYLVSQIYNKNVVFNLVNLKKMHLNSD

IFTQAVSLKLKNRENRLYRVLKKSLNKVKLPNISIIKEKSLAHGSGNKNNNEQLLNKLRNLKINSLFTFNSAFARRDS

LNKLLLNIFPYALDQQVLATPQAGNFKKEIKSRRSLASVFEERKEYPVSLRNYILRSLKHMKIRGIRIEAKGRLTRRF

TASRSVFKMKWKGGLKNVDSSFKGLSAVILRGHVKSNVQYSMLHSRNRNGAFGVKGWVSSKDYN"

8951 9021 gene

gene trnT(tgt)

8951 9021 tRNA

product transfer RNA Threonine

anticodon (pos:8983..8985,aa:t)

9151 9223 gene

gene trnE(ttc)

9151 9223 tRNA

product Glutamate

anticodon (pos:9184..9186,aa:e)

9256 9326 gene

gene trnM(cat)

note copy 1

9256 9326 tRNA

product transfer RNA Methionine

anticodon (pos:9288..9290,aa:m)

9386 9458 gene

gene trnM(cat)

note copy 2

9386 9458 tRNA

product transfer RNA Methionine

anticodon (pos:9419..9421,aa:m)

9482 9563 gene

gene trnL(taa)

9482 9563 tRNA

product transfer RNA Leucine

anticodon (pos:9516..9518,aa:l)

9758 9829 gene

gene trnA(tgc)

9758 9829 tRNA

product transfer RNA Alanine

anticodon (pos:9791..9793,aa:a)

9895 9967 gene

gene trnF(gaa)

9895 9967 tRNA

product transfer RNA Phenylalanine

anticodon (pos:9928..9930,aa:f)

10156 10238 gene

gene trnL(tag)

10156 10238 tRNA

product transfer RNA Leucine

anticodon (pos:10190..10192,aa:l)

10657 10730 gene

gene trnQ(ttg)

10657 10730 tRNA

product transfer RNA Glutamine

anticodon (pos:10691..10693,aa:q)

10940 11012 gene

gene trnH(gtg)

10940 11012 tRNA

product transfer RNA Histidine

anticodon (pos:10973..10975,aa:h)

11671 11743 gene

gene trnM(cat)

note copy 3

11671 11743 tRNA

product transfer RNA Methionine

anticodon (pos:11705..11707,aa:m)

12697 12768 gene

gene trnC(gca)

12697 12768 tRNA

product transfer RNA Cysteine

anticodon (pos:12730..12732,aa:c)

1. LGKQ01002547.1

>Feature LGKQ01002547.1 Table

239 727 gene

gene atp9_t

239 727 CDS

product truncated ATP synthase F0 subunit c, partial

translation: "MIQAAKIIGTGLATTGLIGAGVGIGVXXXXXXXXXXXNPSLRGQLFSYA

ILGFAFAEATDLFALSFLSVWIKTKTQLQKHTFQILALSTSPYILCSYSPVFLLTPLHSLGSSLKPMNFLTVNACYRA

FSFSTLSSTKLMFNNSLVVWNKNQTALIIVNINLNL"

Atp9_t gene is on two separate contigs (LGKQ01002547.1 and LGKQ01002140.1). Likely due to assembly issue a total of 3 amino acids are missing in the assembly.

1. LGKQ01002140.1

>Feature LGKQ01002140.1 Table

>0 1415 gene

gene atp9_t

>0 1415 CDS

product truncated ATP synthase F0 subunit c, partial

translation: "RAFSSYSVSSTKRMVNNSLVVWSKRHTVLIHRVDIKPNLMLITLKARNFSATPSLSSLNKKK

LLVIFSKENFLKHFSKKNVCLGLFMIIIIALIKCSGIAEIILLFLFHSNPEWAQLALSSALGLPLRLVLKGVLDAIFD

GVNFDGFDDNWNRMTMGEDLPYNKPQPKQIEHKKSPYLLKSGDEGEPISRQDKGKGRAVDSPTLVQATGQGQATGQGQ

ITDNNPGQSSSGISTFGRENGITYTDGDVSVNINIMAPYQLAQGVQKKGWDFLLNILENSLSSGKVTEMVEICFTTQN

YNGDKYLMGMRFDLRTLPLNHSLHPDNNSSIFSLIDKTNARTITKHIIPIEDTIEKFVAHLWSENSKLAGRRSNEDVK

LNIDKYPNVLGSIPENLKIGNLLRQQDNNLGNNASSNNQNTRAQSSNTQVTQQQSQGLQSSRNSNVSGNSNFDTSILG

KRKRSYSTSSRLYIPAQA"

1. LGKQ01002289.1

>non-coding DNA
