# Supplementary material for: The First Mitochondrial Genome of Ciborinia camelliae and Its Position in the Sclerotiniaceae Family
Source: Front Fungal Biol. 2022 Feb 9;2:802511. doi: 10.3389/ffunb.2021.802511 (PMC10512376; doi:10.3389/ffunb.2021.802511)
Supplement: Supplementary file 6 [file Data_Sheet_6.docx]

**Linear regression and Pearson’s correlation analysis**

We investigated the association between the mitogenome size (dependent variable) and the number of introns, non-conserved ORFs and tandem repeats (independent variables).

The analysis was performed considering the following species: *Botryotinia fuckeliana* (KC 832409), *Ciboria shiraiana* (CM 017871.1), *Ciborinia camelliae* strain ITAC2 mitogenome (OK326902), *Monilinia fructicola* (NC_056195.1), *Monilinia laxa* (NC_051483.1), *Sclerotinia borealis* (KJ434027), *Sclerotinia sclerotiorum* (KT283062) and *Glarea lozoyensis* (NC_031375.1).


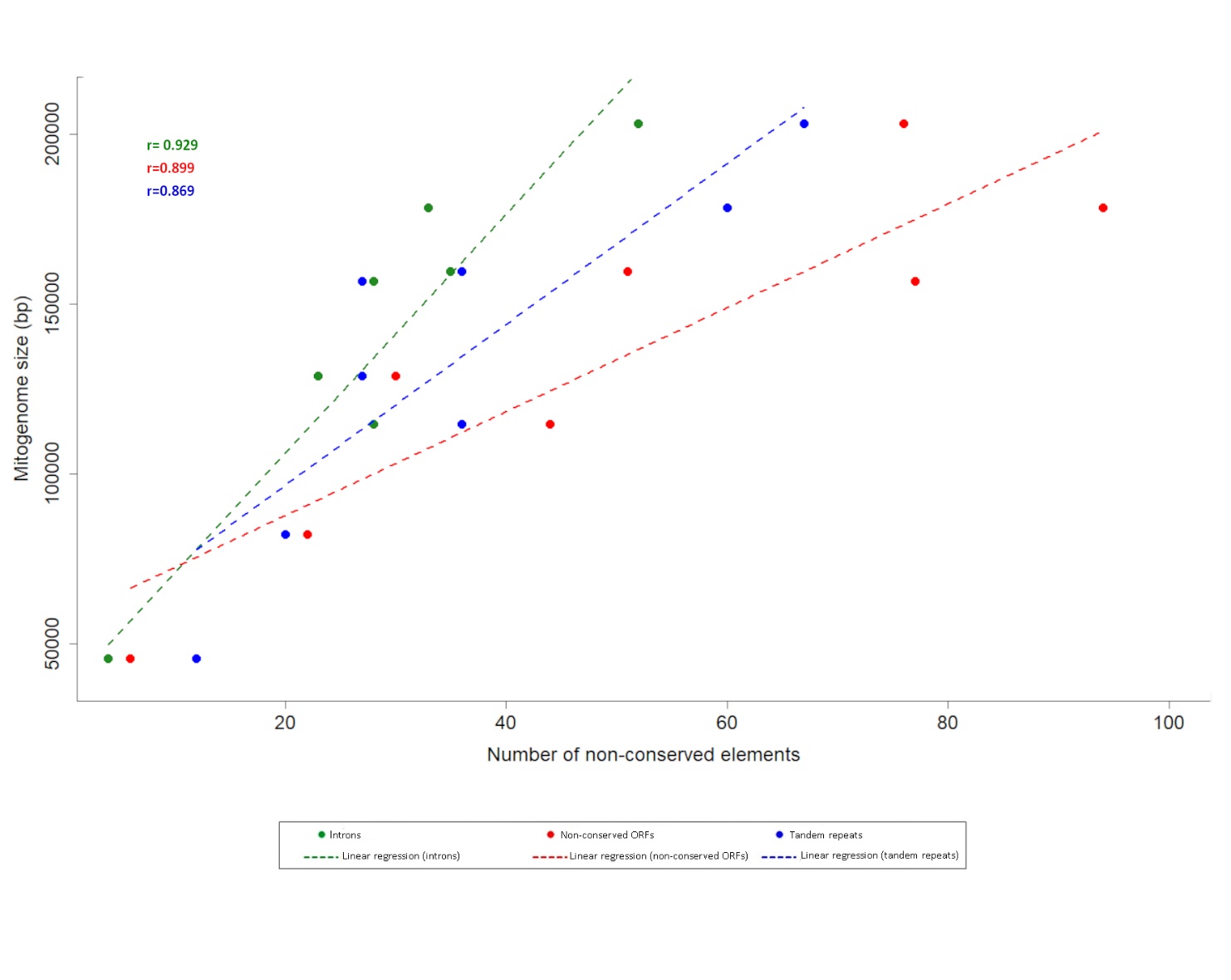


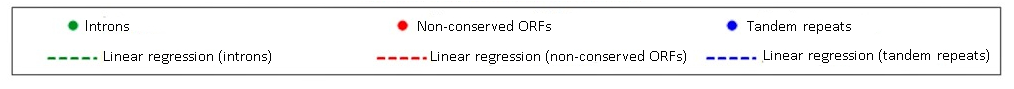


r is the Pearson’s coefficient.

| **Independent variable** | **Pearson’s coefficient** | **p-value** |
| --- | --- | --- |
| Number of introns | 0.929 | 0.0008401 |
| Number of non-conserved ORFs | 0.899 | 0.002354 |
| Number of tandem repeats | 0.869 | 0.005025 |
