# Supplementary material for: The First Mitochondrial Genome of Ciborinia camelliae and Its Position in the Sclerotiniaceae Family
Source: Front Fungal Biol. 2022 Feb 9;2:802511. doi: 10.3389/ffunb.2021.802511 (PMC10512376; doi:10.3389/ffunb.2021.802511)
Supplement: Supplementary file 7 [file Data_Sheet_7.docx]

Supplementary file 1

ORFs analysis of *C. camelliae* mitogenome.

Supplementary file 2

Comparative codon usage of Sclerotiniaceae mitogenomes

Supplementary file 3

Comparative Gene by gene codon usage in Sclerotiniaceae mitogenomes

Supplementary file 4

Codon usage comparison in *C. camelliae* mt genes and ORFs

Supplementary file 5

Protein sequences of the *C. camelliae* ICMP 19812 mitogenome

Supplementary file 6

Association between the mitogenome size and the number of introns, non-conserved ORFs and tandem repeats in Sclerotiniaceae.
